# Alteration of a Shiga toxin-encoding phage associated with a change in toxin production level and disease severity in *Escherichia coli*

**DOI:** 10.1099/mgen.0.000935

**Published:** 2023-02-23

**Authors:** Tatsuya Miyata, Itsuki Taniguchi, Keiji Nakamura, Yasuhiro Gotoh, Dai Yoshimura, Takehiko Itoh, Shinichiro Hirai, Eiji Yokoyama, Makoto Ohnishi, Sunao Iyoda, Yoshitoshi Ogura, Tetsuya Hayashi

**Affiliations:** ^1^​ Department of Bacteriology, Graduate School of Medical Sciences, Kyushu University, Fukuoka 812-8582, Japan; ^2^​ Department of Pediatrics, Graduate School of Medical Sciences, Kyushu University, Fukuoka 812-8582, Japan; ^3^​ School of Life Science and Technology, Tokyo Institute of Technology, Meguro, Tokyo 152-8550, Japan; ^4^​ Division of Bacteriology, Chiba Prefectural Institute of Public Health, Chiba 260-8715, Japan; ^5^​ Center for Emergency Preparedness and Response, National Institute of Infectious Diseases, Musashi-Murayama, Tokyo 208-0011, Japan; ^6^​ Department of Bacteriology I, National Institute of Infectious Diseases, Shinjuku, Tokyo 162-8640, Japan; ^7^​ Division of Microbiology, Department of Infectious Medicine, Kurume University School of Medicine, Kurume, Fukuoka 830-0011, Japan

**Keywords:** disease severity, genome analysis, population structure, STEC O157:H7 clade 8, Stx2 production level, Stx2a phage subtype

## Abstract

Among the nine clades of Shiga toxin (Stx)-producing *Escherichia coli* O157:H7, clade 8 is thought to be highly pathogenic, as it causes severe disease more often than other clades. Two subclades have been proposed, but there are conflicting reports on intersubclade differences in Stx2 levels, although Stx2 production is a risk factor for severe disease development. The global population structure of clade 8 has also yet to be fully elucidated. Here, we present genome analyses of a global clade 8 strain set (*n*=510), including 147 Japanese strains sequenced in this study. The complete genome sequences of 18 of the 147 strains were determined to perform detailed clade-wide genome analyses together with 17 publicly available closed genomes. Intraclade variations in Stx2 production level and disease severity were also re-evaluated within the phylogenetic context. Based on phylogenomic analysis, clade 8 was divided into four lineages corresponding to the previously proposed SNP genotypes (SGs): SG8_30, SG8_31A, SG8_31B and SG8_32. SG8_30 and the common ancestor of the other SGs were first separated, with SG8_31A and SG8_31B emerging from the latter and SG8_32 emerging from SG8_31B. Comparison of 35 closed genomes revealed the overall structure of chromosomes and pO157 virulence plasmids and the prophage contents to be well conserved. However, Stx2a phages exhibit notable genomic diversity, even though all are integrated into the *argW* locus, indicating that subtype changes in Stx2a phage occurred from the γ subtype to its variant (γ_v1) in SG8_31A and from γ to δ in SG8_31B and SG8_32 via replacement of parts or almost entire phage genomes, respectively. We further show that SG8_30 strains (all carrying γ Stx2a phages) produce significantly higher levels of Stx2 and cause severe disease more frequently than SG8_32 strains (all carrying δ Stx2a phages). Clear conclusions on SG8_31A and SG8_31B cannot be made due to the small number of strains available, but as SG8_31A (carrying γ_v1 Stx2a phages) contains strains that produce much more Stx2 than SG8_30 strains, attention should also be paid to this SG.

## Data Summary

All supplementary figures and supplementary tables, as well as the main figures and tables, are provided in the Microbiology Society’s figshare account 10.6084 /m9.figshare.21514572 (https://doi.org/10.6084/m9.figshare.21514572.v1) [1]. The raw read sequences and closed genome sequences generated for this study have been deposited in GenBank/EMBL/DDBJ under the BioProject accession number PRJDB13729 and the BioSample accession numbers SAMD00495861–SAMD00496014 (see also Table S1, available with the online version of this article).

**Impact Statement**

Shiga toxin (Stx)-producing *Escherichia coli* (STEC) is an important intestinal pathogen worldwide. STEC includes strains of various serotypes, and O157:H7 is most frequently associated with human disease. Clade 8 of O157:H7 is thought to be highly pathogenic and cause severe disease such as haemolytic uraemic syndrome more often than other clades. Although two subclades have been proposed, its global population structure has not yet been fully analysed. In addition, conflicting data exist on differences between the subclades regarding the production level of Stx2, a subtype of Stx, the production of which is a risk factor for severe disease development. This study involved genome analyses of a global strain set of clade 8, including 35 closed genomes, and re-evaluation of intraclade variations in Stx2 production level and disease severity within the phylogenetic context. We reveal the evolutionary history and current population structure of this important clade of O157:H7, a clear relationship of the Stx2 production level of clade 8 strains with phylogeny and Stx2a phage subtype, and a higher risk of one lineage (SG8_30) causing severe disease. Our data also call attention to a lineage named SG8_31A, which contains strains that produce much more Stx2 than other strains.

## Introduction

Shiga toxin-producing *Escherichia coli* (STEC) is one of the most important intestinal pathogens in many industrial counties and causes mild to bloody diarrhoea and occasionally life-threatening haemolytic uraemic syndrome (HUS) [2]. As ruminants such as cattle are the major reservoir, food and water contaminated by the faeces of these animals are the main sources of STEC infection, and due to the extremely low infectious dose required for infection (estimated to be<100 cells), STEC has caused many large outbreaks [2, 3]. Typical STEC strains possess a pathogenicity island called the locus of enterocyte effacement (LEE), which encodes a type 3 secretion system (T3SS) [3, 4], multiple lambda-like prophages (PPs) that encode T3SS effectors [5], and a plasmid that encodes multiple potential virulence factors, such as enterohaemolysin [3, 4]. While the T3SS is required for robust adhesion of STEC to epithelial cells and to cause diarrhoea [4, 6], Shiga toxins (Stxs) are the key virulence factors of STEC to induce HUS [2, 3, 7]. Indeed, only strains that produce Stx are associated with HUS [8].

Stxs belong to the AB_5_-type toxin family [9]. They are classified into two subtypes, Stx1 and Stx2, and both subtypes include several variants (Stx1a, Stx1c and Stx1d; Stx2a–Stx2k) [10–13]; all known Stxs are encoded by PP genomes integrated into the STEC chromosome. STEC can produce Stx1 or Stx2 alone or both in various combinations [9], but the production of Stx2, especially Stx2a, is the most important risk factor for HUS development, regardless of the STEC serotype [14–16].

Among the seven major STEC serogroups (O157, O26, O45, O103, O111, O121 and O145), O157:H7 is most frequently associated with human disease worldwide [2]; these strains carry *stx1a*, *stx2a* or *stx2c*, alone or in combination [17]. Manning *et al.* divided O157:H7 STEC strains into nine clades based on 96 SNPs, and each clade was further divided into several SNP genotypes (SGs) [17]. Among the nine clades, clade 8 has been proposed to be a highly pathogenic clade based on epidemiological data [17, 18]. Clade 8 strains harbour *stx2a* or a combination of *stx2a* and *stx2c*, though most clade 8 strains are negative for *stx1a* [17]. In a previous study, we analysed the correlation of the variation in Stx2a phage genomes with the Stx2 production level of various O157:H7 strains, including clade 8 strains. We defined six subtypes of Stx2a phages (α, β, γ, δ, ε and ζ) based on the structural variation in their early regions and showed a significant correlation between the Stx2a phage subtype and Stx2 production level of host strains [19]. In addition, the clade 8 strains analysed (*n*=14) were divided into two subclades (referred to as subclades 8a and 8b) by core-genome-based phylogenetic analysis. All subclade 8a strains, which include TW14359 [17, 20] and EC4115 [21], both isolated in the ‘2006 spinach outbreak’ in the USA, carry γ Stx2a phages that confer the ability to produce the highest level of Stx2 among the four major Stx2a phage subtypes (α, β, γ and δ). In contrast, all subclade 8b strains carry δ Stx2a phages, which are associated with a lower Stx2 production level. Accordingly, the Stx2 production level of the subclade 8a strains is higher than that of subclade 8b strains. Therefore, the proposed high virulence potential of clade 8 may be explained, at least in part, by the presence of subclade 8a, which has acquired the γ subtype of the Stx2a phage. Nevertheless, Hirai *et al.* [22] reported a contrasting observation that subclade 8b strains produce significantly more Stx2 than subclade 8a strains. Although it is unknown why such disparate data were obtained, most strains analysed in the two studies were isolated in restricted regions of Japan and different protocols for sample preparation were employed. Furthermore, as the global population structure of clade 8 strains has yet to be fully elucidated, it is unknown whether these strains represent the entire clade 8 population.

In this study, we performed whole-genome-sequence-based phylogenetic analysis of 510 clade 8 strains from various geographical regions and isolation sources to show their global population structure, and analysed 35 closed clade 8 genomes to understand their genomic diversity, particularly that of their PPs, other integrative elements (IEs) and plasmids. Furthermore, using a strain set that represents the entire clade 8 population as much as possible, we analysed Stx2 production levels, Stx2a phage subtypes and clinical records of clade 8 strains to re-evaluate the relationship between the Stx2 production levels of clade 8 strains, their phylogeny and subtypes of Stx2a phages they carry. We further analysed differences in the risk of severe disease development between lineages in clade 8 identified by whole-genome-sequence-based phylogenetic analysis.

## Methods

### Dataset

We sequenced 154 clade 8 strains isolated in Japan, but 7 genomes were not used due to low sequence quality. Of the 147 genome sequences of Japanese strains in the final dataset, 18 were closed as described in the next subsection. We confirmed that they belonged to clade 8 using the SNP set defined by Manning *et al.* [17] and a clade 8-specific SNP (ECs2357 C539A) [23]. To collect clade 8 genomes available in public databases, among the O157:H7 closed genomes in the National Center for Biotechnology Information (NCBI) database (*n*=93, access date 20/07/2019) and O157:H7 draft genomes in EnteroBase (*n*=3138, access date 27/01/2020), we selected strains carrying the clade 8-specific SNP (ECs2357 C539A) by blastn search [24] for closed genomes or by read mapping using bwa-mem [25] and SNP detection using SAMtools mpileup [26] for draft genomes. After removing low-quality genomes and deduplicating genomes having identical core sequences, the final dataset comprised 35 closed and 475 draft genomes (see Fig. S1 for more details of the selection criteria and processes and Table S1 for the full set of strain information). The accession numbers of each genome are shown in Table S1.

### Genome sequencing and assembly

Genomic DNA of the strains sequenced in this study was extracted and purified using Genomic-tips 100/G and a genomic DNA buffer set (Qiagen) from overnight cultures in lysogeny broth (LB) at 37 °C following the manufacturer’s protocol, with minor modifications including the addition of SDS (final concentration 1 %) after adding buffer B2 and incubation for 1 h at 50 °C. For short-read sequencing, libraries were prepared using the Nextera XT DNA sample prep kit (Illumina) or NEBNext Ultra II FS DNA library preparation kit (New England Biolabs) and sequenced using the Illumina MiSeq platform to generate paired-end sequence reads (301 bp ×2). Reads were trimmed by Platanus trim [27] with default parameters and assembled by Platanus_B_v1.3.1 [28]. Scaffolds ≥300 bp were used in this study.

To determine complete genome sequences, size selection of purified DNA was performed using magnetic beads (AMPure XP; Beckman Coulter) to obtain longer DNA fragments. Sequencing libraries were prepared using a rapid barcoding kit (SQK-RBK004), sequenced using the R9.4.1 flow cell with the Oxford Nanopore Technologies (ONT) MinION platform, and base-called using Guppy GPU ver. 3.4.5. (ONT). Reads were trimmed using NanoFilt [29] with the following parameters: minimum length=7 000 bp, minimum quality score=10 and 5'-terminal 100 bases cutting. For sequencing of three strains (93_161312, CEC13091 and F690), only≥15 kb reads were used for assembly to gain better results. For sequencing of strain F765, the minimum length was changed to 2000 bp to salvage its small plasmid sequence. ONT read assembly and polishing were performed using the microPIPE pipeline [30]. In brief, trimmed ONT reads were assembled using Flye (v2.8.3) [31] with the option ‘--plasmids’ and polished with ONT reads using four iterations of Racon (v1.4.20) [32] followed by one iteration of Medaka (v1.4.3) (GitHub – https://github.com/nanoporetech/medaka). Output contigs were further refined with Illumina short reads using NextPolish (v1.3.1) (GitHub – https://github.com/Nextomics/NextPolish). As the chromosome of strain 93_161312 and the plasmids of strain F690 were not circularized, manual curation was performed using Minimap2 [33], Integrative Genomics Viewer (igv) [34] and GenomeMatcher v3.0.2 [35] to obtain their circular chromosome and plasmid sequences.

### Phylogenetic analyses and strain clustering

Using the raw Illumina reads of 475 draft genomes and the chromosome sequences of 35 closed genomes as queries, core-genome SNPs were identified by BactSNP [36]. The depth and allele frequency thresholds used were 5 and 0.8, respectively, and the clade 8 strain TW14359 was used as a reference. After removing recombinogenic SNPs with Gubbins [37], a maximum-likelihood (ML) phylogenetic tree was reconstructed using RAxML [38] based on the 7477 informative sites with the GTR-GAMMA model of nucleotide substitution and 1000 bootstraps. Strain Sakai (accession no. BA000007.3), which belongs to O157:H7 clade 1, was used as an outgroup. The ML tree was displayed using iTOL [39]. Genomic clusters of strains were assigned using fastbaps v.1.0.8 [40].

### Temporal analyses

Timed phylogeny was reconstructed as previously described [41], with some modifications. By excluding 84 genomes lacking temporal information, 426 clade 8 genomes and an outlier [that of O157:H7 strain Sakai (clade 1)] were selected and an ML tree was reconstructed by the same method as described above. Based on this information, the strain set was downsized to 208 genomes using Treemmer v.0.3 [42] with RTL (relative tree length) option of 0.85. The 207 selected strains (other than strain Sakai) are indicated in Table S1. Using the recombination-free SNP sites in the core genome of these 208 genomes, an ML tree was generated again, and the temporal signal in the tree was examined using TempEst [43] by assessing the positive linear relationship between the root-to-tip distance and the year of isolation. The GTR substitution model with the relaxed clock and constant population size model was selected as the best-fit model by assessing the Bayes factor test. The result was summarized as a maximum clade credibility tree using TreeAnnotator in beast v.1.8.4 [44] and visualized with FigTree v1.4.4 (http://tree.bio.ed.ac.uk/software/figtree/).

### SG assignment

Assignment of each strain to the SG was performed according to the scheme proposed by Manning *et al.* [17], with clade 8 strains divided into five SGs (SG8_30–SG8_34) based on five SNPs: 1 984 857 G>A and 3 599 366 C>T (unique to SG8_32. 33 and 34), 2 294 999 C>T (unique to SG8_34), 3 838 445 C>T (unique to SG8_30) and 5 398 532 C>T (unique to SG8_33) (nucleotide numbers correspond to those of the Sakai genome, see Table S2 for the SNP information of each SG). These SNPs were identified by BactSNP as described above, and the old version of the Sakai genome (accession no. BA000007.2) was used as a reference (see Table S1 for SGs of each strain analysed).

### *stx* genotyping

*stx* operons in the closed genomes were subtyped by aligning their sequences against the previously published collection of *stx* operons [10]. The Stx subtypes of 17 strains were clearly determined, as their *stx* operons showed exact sequence matches to some reference *stx* operons in the collection. Although the remaining 18 genomes showed no exact match, they were typed as *stx2a* because their *stx* operons differ from one of the reference *stx2a* operons only by one synonymous SNP (129 G>A).

For detection and subtyping of *stx1* in draft genomes, trimmed reads were mapped to the *stx1a* operons of four O157:H7 strains [Sakai, clade 1; FDAARGOS_293 (accession no. CP022050.2), clade 4/5; PV15-279 (AP018488.1), clade 9; and 180-PT54 (CP015832.1), clade 7] by bwa-mem with default parameters. SAM files of top-hit reads to each reference were entered into a custom script (GitHub – https://github.com/IEkAdN/Type) to report ‘homology’ and ‘coverage’. The homology value was determined by calculating the proportion of exact matches at each base position in each reference sequence using top-hit reads and averaging the values across the reference sequence covered by top-hit reads. The coverage value represents the proportion of sequences covered by top-hit reads in the reference sequence. As the sequence similarities between the *stx1a* and *stx1c* operons and between the *stx1a* and *stx1d* operons in the reference collection [10] were calculated to be 96 and 91 %, respectively, we defined a strain as possessing an *stx1* variant when its homology and coverage were both≥99 %. *stx1* variants other than *stx1a* were not detected in the dataset analysed in this study even with lower thresholds.

*stx2* operons in draft genomes were examined and subtyped using the same strategy. Because two different *stx2* operon sequences were found for each of the *stx2a* or *stx2c* operons in the closed clade 8 genomes, four *stx2* operons were used as references representing each sequence type: *stx2a* from strains TW14359 (SG8_30) and 08–3918 (SG8_33) and *stx2c* from strains TW14359 (SG8_30) and 08–3914 (SG8_31). However, the intersubtype nucleotide sequence identities between these *stx2a* and *stx2c* operons were≥98.2 %; those between the *stx2a* and *stx2c* operons in the reference collection [10] were≥97.4 %, leading to frequent cross-mapping of reads between the two *stx2* subtypes. Therefore, exact-match reads were selected by the BamTools filter [45] using the option ‘NM:0’, and *stx2* subtypes were assigned when such exact-match reads covered the entire sequence of any of the four references (100 % coverage). Results with any ambiguity were checked by manual inspection using igv. Note that any SNPs indicating the presence of *stx2* subtypes other than *stx2a* and *stx2c* were not detected, and that short-read assembly is often unable to obtain full-length *stx* operons when a genome contained both *stx2a* and *stx2c* operons or multiple *stx2a* operons owing to high sequence similarity.

### Identification and analysis of PPs and IEs in closed genomes

The chromosome sequence of strain TW14359 was reannotated by dfast [46], and its PP regions were predicted by phaster [47, 48], followed by manual curation to precisely identify each PP region, including *attL* and *attR* sequences. IEs not detected by phaster were identified by searching genes annotated as ‘integrase genes’, followed by manual inspection. For the other closed genomes, PP/IE integration sites (*attB* sites) identified in strain TW14359 were analysed for the presence of PPs/IEs and their sequences, if present. PPs/IEs not found in strain TW14359 were identified by integrase gene search, as described above. The PPs/IEs found in all closed genomes (Table S3) were annotated by dfast, followed by manual curation. Insertion sequences (ISs) in the PP/IE sequences were detected and typed by ISfinder [49]. Genetic organizations of PPs/IEs were visualized by GenomeMatcher v3.0.2. Sequence similarity of PPs/IEs located at the same loci was analysed by dot-plot analyses using GenomeMatcher v3.0.2 and by calculating pairwise Mash distances [50] with default parameters (*k*-mer size of 21, and sketch size of 1000). The results of pairwise Mash distance analysis are presented as violin plots using RAWGraphs 2.0 beta (https://dl.acm.org/doi/10.1145/3125571.3125585).

### Detection of plasmid replicons and antimicrobial-resistance (AMR) genes

Plasmid replicons were detected by PlasmidFinder v2.1 [51] with a threshold of≥80 % identity and≥60 % coverage. As the pcolD157 [52] replicon found in strain F765 is not included in the PlasmidFinder database, it was detected by blastn with a threshold of≥90 % identity and≥90 % coverage. AMR genes were detected by ABRicate v1.0.1 (GitHub – https://github.com/tseemann/abricate) using ResFinder [53] as a database, with a threshold of≥80 % identity and≥60 % coverage.

### Stx2a phage subtyping

Subtypes of Stx2a phages in the closed genomes were determined by two-step *in silico* PCR (GitHub – https://github.com/bowhan/kent/tree/master/src/isPcr) with default conditions, except for the maximum PCR product size in the first PCR (15 000 bp), using the sequences of the six previously defined subtypes [19] as references. Stx2a phages in five genomes, which were assigned to the γ subtype by the first PCR but negative in the second PCR, were assigned as a γ variant (named subtype γ_v1). Subtypes of Stx2a phages in the draft genomes were determined by the read mapping strategy described for *stx* subtyping using the nucleotide sequences of the first PCR regions of six Stx2a phage subtypes and subtype γ_v1 with the≥97 % homology and≥98 % coverage threshold.

### Measurement of Stx2 production

Overnight cultures of strains to be examined were inoculated into 2 ml LB medium at an OD_600_ of 0.1 and incubated at 37 °C with shaking. When the OD_600_ reached 0.7–0.8, mitomycin C (Kyowa Hakko Kirin or Wako Yakuhin) was added to the culture at a final concentration of 500 ng ml^−1^. After further incubation for 5 h, 100 µl of the culture was subjected to sonication for 4 min at 250 W (Bioruptor UCD-250; Cosmo Bio), followed by centrifugation (7 700 ***g*** for 10 min at 4 °C) to obtain the supernatant. The Stx2 concentration in each supernatant was determined by the fluorescence resonance energy transfer (FRET) system we previously described [54], except that we used Verotoxin-2 (Nacalai Tesque) as the Stx2 standard. Mean values of three biological duplicates were used for comparison of Stx2 production levels between strains.

### Statistical analyses

The Kruskal‒Wallis test followed by Bonferroni’s correction was used for inter-SG comparison of Stx2 production levels and the prevalence of *stx* genotypes and Stx2a phage subtypes. Fisher’s exact test was used for comparison of incident rates of severe disease (defined as bloody diarrhoea or HUS) between SGs and between Stx2a phage subtypes. All *P* values were two sided, and *P* values <0.05 were considered statistically significant. All statistical analyses were performed with the EZR package (Y. Kanda, Saitama Medical Centre, Jichi Medical University, Saitama, Japan; https://www.jichi.ac.jp/saitama-sct/SaitamaHP.files/statmedEN.html; 2012) in R (R Core Team; 2021).

## Results

### Strain set

The strain set analysed (*n*=510) comprised the 147 Japanese isolates sequenced in this study and 363 strains with genome sequences obtained from public databases (see Table S1 for detailed information of each strain, and Methods and Fig. S1 for strain selection). Of the 510 genomes, 35 were closed, including 18 genomes determined in this study, such that closed genomes covered the entire clade 8 population as much as possible.

As summarized in Table 1, the 510 strains were isolated in 10 countries, and 92.5 % were from 4 of the countries, the USA (42.5 %), Japan (29.4 %), New Zealand (10.4 %) and the UK (10.2 %). The main source of strains was humans (54.7 %), followed by bovines (22.7 %). The remaining 14.0 % were derived from other animals, food, feed or environmental sources. Most strains (95.5 %) carry *stx2a*; 215 (42.2 %) harbour *stx2a* only, 272 (53.3 %) additionally carry *stx2c* (*stx2a*/*stx2c*) and 5 (1.0 %) *stx1a* along with *stx2a* and *stx2c* (*stx1a*/*stx2a*/*stx2c*). Only 2.7 % carry *stx2c* alone. This proportion of *stx* genotypes is similar to that previously reported (*stx2a*, 42.4 %; *stx2a/stx2c*, 57.6 %; and *stx1-*negative, 96.3 %) [17].

**Table 1.** Summary of the strains analysed in this study

Characteristic		SNP genotype			
	Total	8_30	8_31A	8_31B	8_32
**Country**					
Japan	150 (29.4 %)	107 (38.4 %)	5 (17.9 %)	2 (1.9 %)	36 (36.7 %)
USA	217 (42.5 %)	146 (52.3 %)	13 (46.4 %)	3 (2.9 %)	55 (56.1 %)
Canada	18 (3.5 %)	11 (3.9 %)	1 (3.6 %)	2 (1.9 %)	4 (4.1 %)
UK	52 (10.2 %)	4 (1.4 %)	3 (10.7 %)	43 (41.0 %)	2 (2.0 %)
Other Eur.*	11 (2.2 %)	8 (2.9 %)	2 (7.1 %)	1 (1.0 %)	0
New Zealand	53 (10.4 %)	0	0	53 (50.5 %)	0
Argentina	6 (1.2 %)	2 (0.7 %)	4 (14.3 %)	0	0
No information	3 (0.6 %)	1 (0.4 %)	0	1 (1.0 %)	1 (1.0 %)
**Host**					
Human	279 (54.7 %)	153 (54.8 %)	13 (46.4 %)	52 (49.5 %)	61 (62.2 %)
Animals					
Bovine	116 (22.7 %)	71 (25.4 %)	5 (17.9 %)	30 (28.6 %)	10 (10.2 %)
Other animals	11 (2.2 %)	8 (2.9 %)	0	1 (1.0 %)	2 (2.0 %)
Food/feed					
Beef	20 (3.9 %)	16 (5.7 %)	2 (7.1 %)	0	2 (2.0 %)
Other food/feed	11 (2.2 %)	9 (3.2 %)	1 (3.6 %)	0	1 (1.0 %)
Environment	29 (5.7 %)	5 (1.8 %)	2 (7.1 %)	20 (19.0%)	2 (2.0 %)
No information	44 (8.6 %)	17 (6.1 %)	5 (17.9 %)	2 (1.9 %)	20 (20.4 %)
**stx genotype**					
*stx2a*	215 (42.2 %)	50 (17.9 %)	1 (3.6 %)	69 (65.7 %)	95 (96.9 %)
*stx2c*	14 (2.7 %)	10 (3.6 %)	3 (10.7 %)	1 (1.0 %)	0
*stx2a/stx2c*	272 (53.3 %)	211 (75.6 %)	24 (85.7 %)	35 (33.3 %)	2 (2.0 %)
*stx1a/stx2a/stx2c*	5 (1.0 %)	5 (1.8 %)	0	0	0
Not detected	4 (0.8 %)	3 (1.1 %)	0	0	1 (1.0 %)
**total**	**510**	**279**	**28**	**105**	**98**

*Finland (*n*=2), Italy (*n*=2), Norway (*n*=1) and Denmark (*n*=6).

### Phylogenetic analysis of clade 8 strains

**Fig. 1.**
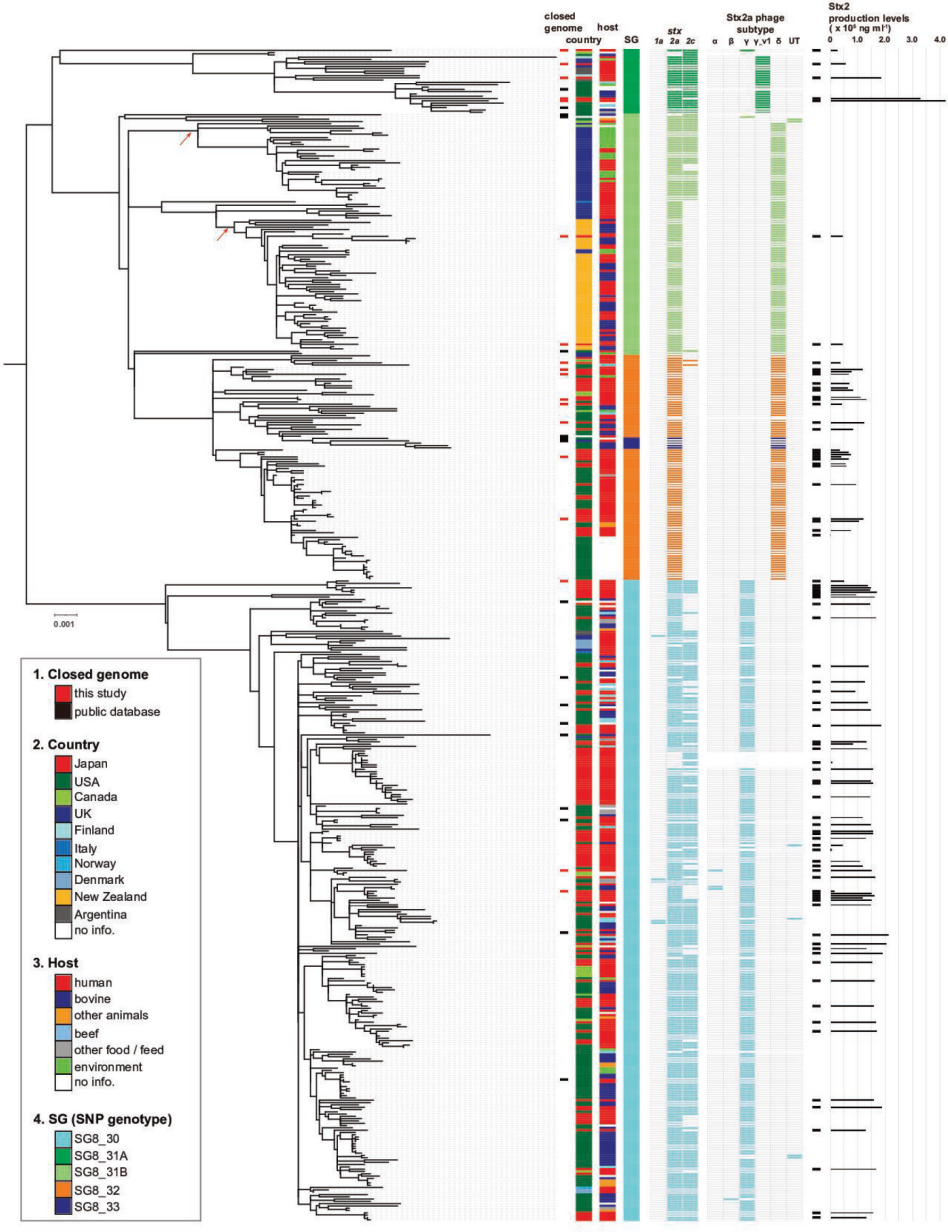
ML phylogenetic tree of the 510 clade 8 strains analysed in this study. The data of *stx* genotypes and Stx2a phage subtypes are coloured according to the SGs of strains. Short black bars beside the column of Stx2 production levels indicate the strains used for the Stx2 production level analysis. UT in the column of Stx2a phage subtypes indicates untypeable. The scale bar indicates nucleotide substitutions per site.

To reveal the evolutionary history and global population structure of clade 8 strains, we performed a core-genome-based phylogenetic analysis of the 510 strains using recombination-free 7477 core-genome SNPs (Fig. 1) and a temporal analysis (Fig. S2). Mapping of SG information of each strain (SG8_30, SG8_31, SG8_32 or SG8_33; note that SG8_34 was not found in the present strain set) to the ML tree (Fig. 1) and the timed phylogeny reconstructed (Fig. S2) revealed that clade 8 was first separated into SG8_30 and the common ancestor of other SGs approximately 96 years ago[95 % highest posterior density (HPD) 99–88 years]. Then, the latter was separated around 1931 (95 % HPD 1929–1940) into two lineages of SG8_31 (referred to as SG8_31A and SG8_31B, respectively; they were analysed separately in this study) and SG8_32 emerged from SG8_31B around 1956 (95 % HPD 1953–1957). SG8_33 represents a sublineage of SG8_32. Therefore, we considered SG8_32 and SG8_33 as a single lineage, simply referred to as SG8_32 in this manuscript. The two subclades proposed for clade 8 (subclades 8a and 8b) in previous studies [19, 22] correspond to SG8_30 and SG8_32, respectively. Note that the result of clustering by fastbaps at level 1 was largely congruent with the SG-based classification, although it generated more clusters (Fig. S3).

Every SG contains strains from various geographical regions, but several sublineages are composed of strains from a single country, such as the two sublineages in SG8_31B indicated by arrows in Fig. 1. They mostly comprise UK (33/34 strains) and New Zealand (53/57 strains) isolates; thus, they can be regarded as UK- and New Zealand-specific sublineages, respectively. Although such region-specific sublineages have not been described in clade 8, country-specific genomic features or population structures of O157:H7 strains in the UK [55] or the USA [56] were previously reported.

The *stx* genotypes exhibit a clear phylogeny-dependent pattern (Fig. 1, Table 1). Most strains in SG8_30 and SG8_31A carry *stx2a*/*stx2c*, and nearly all SG8_32 strains contain only *stx2a*. The SG8_31B strains harbour *stx2a*/*stx2c* or *stx2a* alone, following the phylogeny of each strain, with a few exceptions. This result suggests that the most recent common ancestor (MRCA) of clade 8 strains had already acquired *stx2a* and *stx2c* and that *stx2c* was later lost during the diversification of SG8_31B and upon the emergence of SG8_32 from SG8_31B (Fig. S2).

### Analysis of variations in genome structure and mobile genetic element (MGE) repertoire using complete genome sequences

To better understand the genomic features of clade 8, complete genome sequences are necessary, especially to capture a complete view of the variations in genome structure and the repertoire of MGEs, which are often involved in genome rearrangement and variation in gene content. Therefore, we determined the complete genome sequences of 18 clade 8 strains in this study such that they, together with 17 publicly available closed genomes [20, 21, 57–65], covered the entire clade 8 population (Fig. 1), allowing fine and systematic comparisons of genome structure and MGE repertoire.

#### Chromosomal structure

As summarized in Table 2, the chromosomes of the 35 strains are 5.32–5.64 Mb in size (mean 5.46 Mb). Dot plot analysis of these chromosome sequences revealed the chromosome backbones to be well conserved, despite large inversions found in 9 of the 35 strains (25.7 %; Fig. S4). These inversions occur at different positions but are all centred on the replication terminus and associated with PPs, as previously described [66], indicating that recombination between PPs having similar sequences is the major mechanism of relatively frequent chromosome inversions in clade 8. In addition to inversions, several regions showing notable sequence variations in each chromosome were found. As many of them appear to correspond to PP regions, we performed detailed analysis of PPs and IEs, as described below.

**Table 2.** List of 35 closed genomes analysed in this study

Strain name	SG	Isolation source	Isolation year	Country*	Genome size (bp)	Chromosome size (bp)	pO157		Extra plasmid		Stx genotype	Stx2a phage subtype	Accession no.	Reference
							Size (bp)	Replicons	Size (bp)	Replicon				
CEC13002	8_31A	Human	2013	JP	5 479 241	5 385 437	93 804	FII/FIB	–	–	*stx2a/stx2c*	γ	AP026116/AP026117	This study
53_142304	8_31A	Human	2014	JP	5 481 530	5 386 522	95 008	FII/FIB	–	–	*stx2a/stx2c*	γ_v1	AP026095/AP026096	This study
57_142493	8_31A	Human	2014	JP	5 545 203	5 487 732	57 471	FII	–	–	*stx2a/stx2c*	γ_v1	AP026097/AP026098	This study
06-3462	8_31A	–	–	USA	5 545 237	5 450 250	94 987	FII/FIB	–	–	*stx2c*	Not applicable	CP034794/CP034795	[57]
CEC13004	8_31A	Human	2013	JP	5 517 405	5 422 445	94 960	FII/FIB	–	–	*stx2a*	γ_v1	AP026118/AP026119	This study
93_161312	8_31A	Human	2016	JP	5 6 41 811	5 514 009	94 980	FII/FIB	32 822	IncX4	*stx2a/stx2c*	γ_v1	AP026101/AP026102/AP026103	This study
08-3914	8_31A	–	2007	USA	5 624 664	5 5 29 684	94 980	FII/FIB	–	–	*stx2a/stx2c*	γ_v1	CP034808/CP034809	[57]
28RC1	8_31B	Bovine	1999	USA	5 643 099	5 561 698	81 401	FII/FIB	–	–	*stx2c*	Not applicable	CP015020/CP015021	[58]
2017C-4109	8_31B	–	–	–	5 537 857	5 384 880	93 169	FII/FIB	59 808	IncI2†	*stx2a/stx2c*	γ	CP030767/CP030765/CP030766	[59]
61_150228	8_31B	Human	2014	JP	5 731 303	5 638 999	92 304	FII/FIB	–	–	*stx2a*	δ	AP026099/AP026100	This study
27_141091	8_31B	Human	2014	JP	5 582 377	5 424 430	91 003	FII/FIB	66 944	IncFII(pSE11)	*stx2a*	δ	AP026092/AP026093/AP026094	This study
ATCC 43889	8_31B	Human	–	USA	5 660 288	5 567 434	92 854	FII/FIB	–	–	*stx2a/stx2c*	δ	CP015853/CP015854	[60]
CEC13091	8_32	Human	2013	JP	5 627 247	5 531 748	95 499	FII/FIB	–	–	*stx2a*	δ	AP026108/AP026109	This study
CEC01311	8_32	Human	2001	JP	5 407 703	5 3 24 174	83 529	FII/FIB	–	–	*stx2a*	δ	AP026106/AP026107	This study
CEC01302	8_32	Human	2001	JP	5 425 603	5 331 436	94 167	FII/FIB	–	–	*stx2a*	δ	AP026114/AP026115	This study
F690	8_32	Human	1990s	JP	5 630 853	5 472 504	1 58 349	FII/FIB	–	–	*stx2a*	δ	AP026080/AP026081	This study
F765	8_32	Human	1990s	JP	5 571 929	5 472 415	92 839	FII/FIB	6675	Not detected	*stx2a*	δ	AP026082/AP026083/AP026084	This study
H52_982342	8_32	Human	1998	JP	5 524 294	5 430 116	94 178	FII/FIB	–	–	*stx2a*	δ	AP026085/AP026086	This study
ECP17-46	8_32	Human	–	–	5 569 576	5 475 399	94 177	FII/FIB	–	–	*stx2a*	δ	CP040572/CP040573	[61]
08-3918	8_33	–	–	USA	5 564 973	5 428 647	94 189	FII/FIB	42 137	IncI2 (Delta)	*stx2a*	δ	CP034797/CP034796/CP034798	[57]
272	8_33	Human	2013	UK	5 568 363	5 474 193	94 170	FII/FIB	–	–	*stx2a*	δ	CP018239/CP018240	[62]
CEC03102	8_32	Human	2003	JP	5 553 625	5 481 485	72 140	FII/FIB	–	–	*stx2a*	δ	AP026112/AP026113	This study
CEC08123	8_32	Human	2008	JP	5 489 831	5 395 638	94 193	FII/FIB	–	–	*stx2a*	δ	AP026110/AP026111	This study
CEC96047	8_30	Human	1996	JP	5 449 642	5 390 678	58 964	FII/FIB	–	–	*stx2a/stx2c*	γ	AP026104/AP026105	This study
147	8_30	Livestock	2011	–	5 577 185	5 422 686	94 611	FII/FIB/FIA	59 888	IncI2 (Delta)	*stx2a*	γ	CP028600/CP028602/CP028601	–
2009C-3378	8_30	–	–	USA	5 516 392	5 420 971	95 421	FII/FIB	–	–	*stx2c*	Not applicable	CP034792/CP034793	[57]
SS52	8_30	Bovine	2010	USA	5 583 430	5 488 700	94 730	FII/FIB/FIA	–	–	*stx2a/stx2c*	γ	CP010304/CP010305	[63]
2009C-4687	8_30	–	2009	USA	5 574 764	5 480 158	94 606	FII/FIB/FIA	–	–	*stx2a/stx2c*	γ	CP034799/CP034800	[57]
SS17	8_30	Bovine	2010	USA	5 655 941	5 523 849	94 645	FII/FIB/FIA	37 447	pEC4115	*stx2a/stx2c*	γ	CP008805/CP008806/CP008807	[64]
2010C-3142	8_30	–	2009	USA	5 573 470	5 478 884	94 586	FII/FIB/FIA	–	–	*stx2a/stx2c*	γ	CP034801/CP034802	[57]
EC4115	8_30	Human	2006	USA	5 704 171	5 572 075	94 644	FII/FIB/FIA	37 452	pEC4115	*stx2a/stx2c*	γ	CP001164/CP001163/CP001165	[21]
8_140198	8_30	Human	2014	JP	5 608 759	5 479 903	92 794	FII/FIB/FIA	36 062	IncX4	*stx2a/stx2c*	α + γ	AP026087/AP026088/AP026089	This study
26_141088	8_30	Human	2014	JP	5 526 458	5 430 564	95 894	FII/FIB/FIA	–	–	*stx2a*	γ	AP026090/AP026091	This study
JEONG-1266	8_30	Bovine	2013	USA	5 574 593	5 478 683	95 910	FII/FIB/FIA	–	–	*stx2a/stx2c*	γ	CP014314/CP015816	[65]
TW14359	8_30	Human	2006	USA	5 622 737	5 528 136	94 601	FII/FIB/FIA	–	–	*stx2a/stx2c*	γ	CP001368/CP001369	[20]

∗JP, Japan; USA, United States; UK, United Kingdom.

†Contains the *mcr-1.1* gene.

#### PPs and IEs

**Fig. 2.**
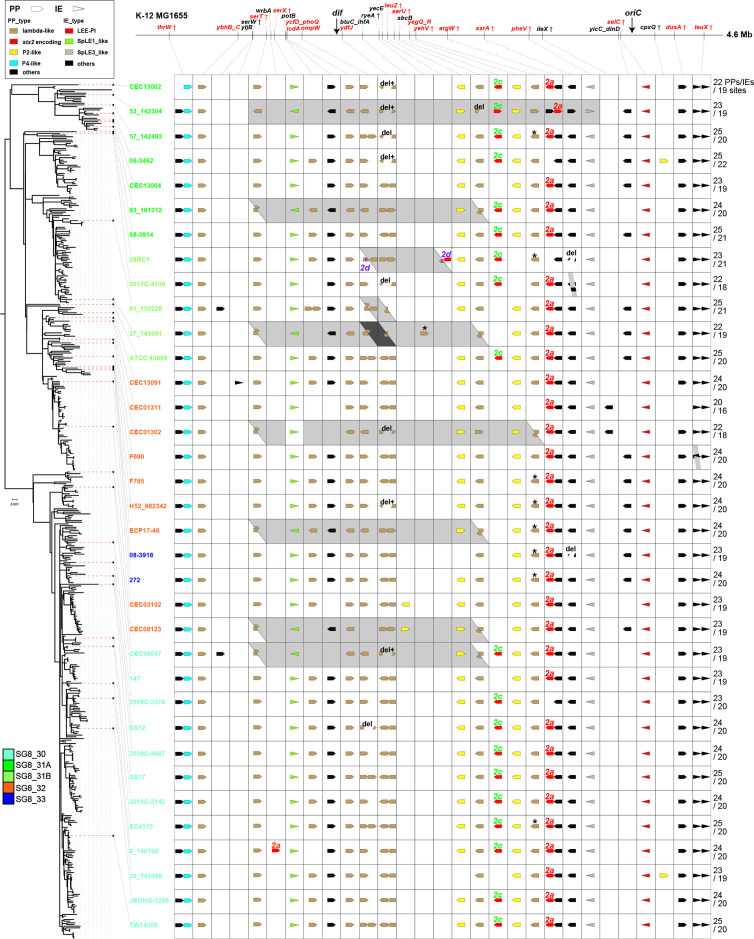
PPs and IEs in 35 closed clade 8 genomes.Strain names are coloured according to their SGs. Integration sites indicated in red are the sites used in most strains (>85 %), and those indicated by daggers are tRNA- or small RNA-encoding genes. Directions of PPs/IEs are denoted according to the direction of chromosome replication. Asterisks indicate the presence of >10 kb insertions. 'del' indicates the presence of deletions and 'del+' indicates the presence of deletions and replacements. Chromosomal inversions are indicated by a grey background. The number of PPs and IEs and the number of their integration sites in each strain are indicated at the rightmost side. The scale bar on the ML tree indicates nucleotide substitutions per site.

As shown in Fig. 2 and listed in Table S3, we identified a total of 29 integration sites of PPs/IEs (24 for PPs and 5 for IEs), including 5 sites where two PPs or two IEs inserted in tandem. Of the 29 integration sites, 18 sites (14 by PPs and 4 by IEs) are occupied by PPs/IEs in most strains (31 or more strains, including all 5 tandem integration sites), accounting for 62 % of the 29 sites. Among the remaining 11 sites, the *sbcB* locus was found to be integrated by all Stx2c phages identified; thus, PP integration at this site was observed only in strains belonging to SG8_30 and SG8_31A and three of the five SG8_31B strains (19 strains in total). At the *yicC_dinD* intergenic region, PP integrations were detected in 15 strains belonging to SG8_31A, SG8_31B or SG8_32 (no SG8_30 strains). In contrast, PP integration at the *potB* locus was observed in all SGs, but only 24 strains in total, suggesting frequent deletions of the PP integrated in *potB* (PP_*potB*) or frequent and repeated acquisition of PP_*potB*. At the other eight sites, PP/IE integration occurred in only one or two strains. The IE found at *serW* (one of the eight sites) in strain CEC13091 (Japanese clinical isolate) carries the SSuT element [67] encoding streptomycin-, sulfonamide- and tetracycline-resistance genes and multiple sets of *cdi* genes for contact-dependent inhibition of growth. This IE is very similar to one found in an O157:H7 clade 7 strain, EC869 (accession no. ABHU01000020) [68], which is also integrated into the *serW* locus (Fig. S5).

**Fig. 3.**
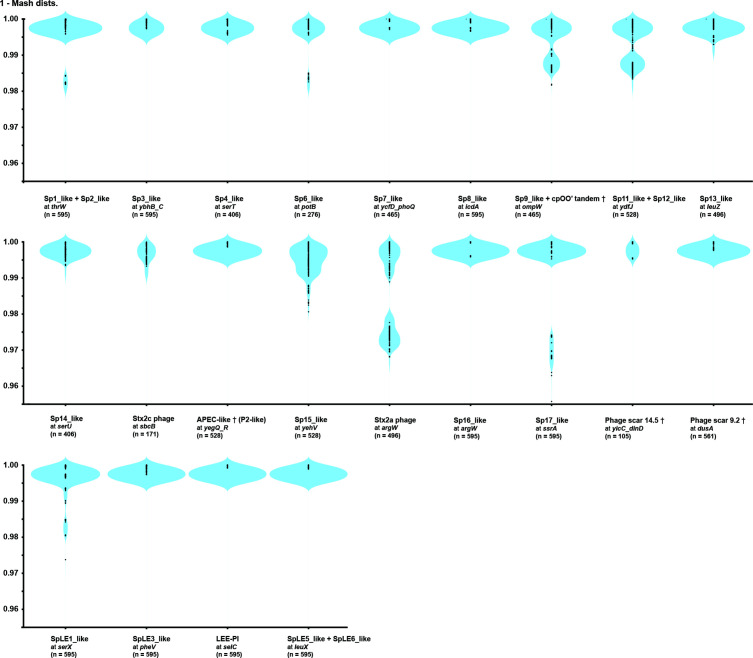
Pairwise Mash distances of the sequences of PPs/IEs integrated into the same locus. Numbers in parenthesis indicate the number of dots. Daggers indicate the PP names used by Eppinger *et al.* [21].

To evaluate the genomic diversity of PPs and IEs integrated into the same locus, we analysed pairwise Mash distances of the PPs and IEs (those integrated into frequently used loci were analysed, 18 for PPs and 4 for IEs; PPs involved in genome inversion were not included). As shown in Fig. 3, the IEs found at the 4 loci and the PPs at 11 loci are highly conserved in sequence, whereas the PPs at 7 loci (at *thrW*, *potB*, *ompW*, *ydfJ*, *yehV*, *argW* and *ssrA*) show certain levels of genomic diversity. The genomes of Stx2c phages (located at *sbcB*) are also highly conserved in sequence, supporting the notion that Stx2c phages were acquired by the MRCA of clade 8 and have been lost in two descendant lineages. This result is consistent with the model that Stx2c phage was acquired by the common ancestor of O157:H7 and maintained by vertical inheritance in the majority of the O157:H7 population [21, 55, 69, 70]. The sequences of the six IEs identified, including two IEs integrated into *leuX* in tandem, are also highly conserved.

To analyse the genomic diversity of the PPs and IEs in more detail, we further performed dot plot analysis (Fig. S6; see Fig. S7 for the genetic structures of representative PPs and IEs integrated at each locus). This analysis revealed that the diversities of the PPs at five loci (*thrW*, *potB*, *ompW*, *yehV* and *ssrA*) observed in pairwise Mash distance analysis are mainly due to deletion of parts of a PP or one of the PPs integrated in tandem (*thrW*, *ompW* and *ssrA*) or the insertion of a PP or PP-like element (*potB* and *yehV*) in a limited number of strains (see Fig. S8 for insertion of an additional PP in strains 61_150228 and EC4115). Eppinger *et al.* [21] reported the presence of a PP-like element inserted in the tRNA-triplet in the Sp15_like PP integrated into *yehV* in strain EC4115. However, the insertion of the second PP apparently occurred by homologous recombination between the resident PP, not by integrase-mediated insertion, because appropriate *attB* sequences were not detected and the order of genes is very unusual if this element was inserted in an integrase-dependent manner. Moreover, the second PP is nearly identical to one of the two PPs integrated in tandem at *ompW* in strain TW14359 (see the lower panel in Fig. S8). At the *ydfJ* locus, where two lambda-like PPs are integrated in tandem, various deletions involving the two PPs, often accompanied by replacement of PP genome segments, have occurred as previously described [71]. The most remarkable sequence diversity was observed between the PPs at *argW* (Stx2a phages), suggesting that replacement of entire or parts of Stx2a phage genomes has occurred in clade 8.

#### Plasmids

As shown in Table 2, all 35 strains carry virulence plasmid pO157 encoding enterohaemolysin and several potential virulence factors. Although the sequences of pO157 plasmids are relatively well conserved, notable differences in length (57–158 kb) were found due to IS-mediated deletion of >10 kb long segments in five strains (Fig. S9a, b) and fourfold amplification of a 20 kb segment in strain F690 (Fig. S9a, c). The 20 kb segment amplification was apparently mediated by IS*1203*, but the precise mechanism and biological significance of this amplification and maintenance are unknown. These IS*1203*-mediated variations in pO157 sequences are mostly strain-specific. In addition, the intact *repE* gene (IncFIA replicon) was detected only in SG8_30 strains, and *repE* was deleted or inactivated by IS*1203* insertion in other strains (Table 2, Fig. S9a, b). A plasmid replicon search of the entire clade 8 strain set confirmed the IncFIA replicon to be conserved in only SG8_30 strains (Fig. S10).

Additional plasmids were found in 9 of the 35 strains (one plasmid in each strain), including an IncI2 plasmid (pMCR-1) in strain 2017C-4109, which carries the colistin-resistance gene *mcr-1.1* [59]. These plasmids vary in size (6.6–66.9 kb) and sequence, though plasmids carrying the same type of replicon share various lengths of nearly identical sequences. These results suggest that gain and loss of various plasmids frequently occurred during the diversification of clade 8, as also shown for STEC O145:H28 [72] and STEC O121:H19 [41]. This notion is supported by results of the plasmid replicon search in the entire clade 8 strain set; 19 types of plasmid replicons were further identified in draft genomes, but their distributions were limited to small numbers of strains (Fig. S10).

#### Horizontally acquired AMR genes

Only two MGEs carrying AMR genes were found in the 35 closed genomes; the above-mentioned IE at *serW* (Fig. S5) in strain CEC13091 [carrying *strA*, *strB*, *tetA(B)* and *sul2* on the SSuT element] [67] and an IncI2 plasmid pMCR-1 (carrying *mcr-1.1*) [59] in strain 2017C-4109. Therefore, we searched for AMR genes in the entire clade 8 strain set. This search detected a total of 18 AMR genes including the above-mentioned 5 genes (Fig. S10, see also Table S1) in 61 strains (12.0 % in the strain set). The number of AMR genes in these strains ranged from one to nine, and 35 strains were considered as multidrug-resistant (MDR) strains as they were genotypically resistant to more than three classes of antimicrobials.

### Analysis of Stx2a phage genomes

**Fig. 4.**
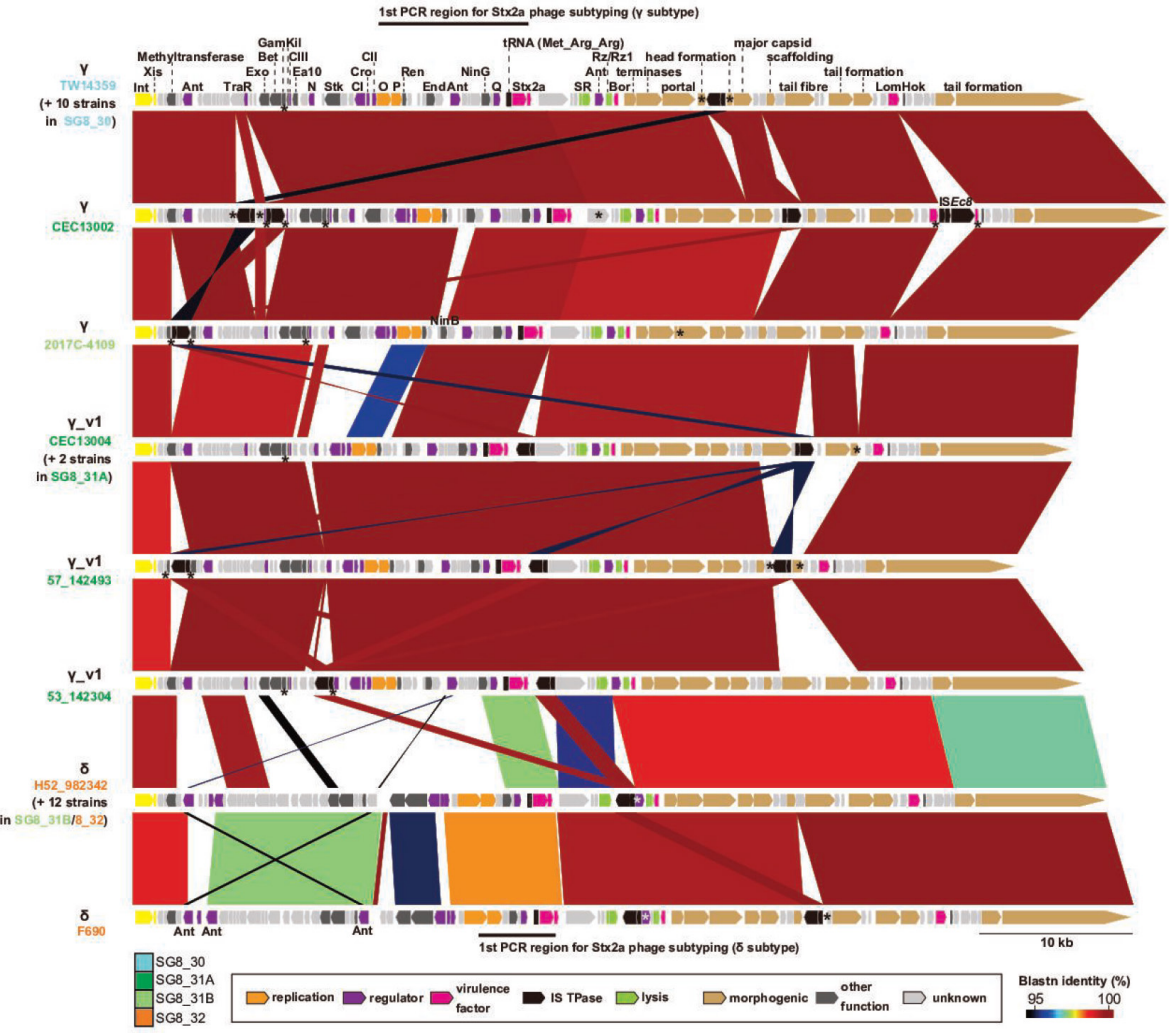
Comparison of genomic structures of Stx2a phages in 32 closed genomes carrying the *stx2a* gene. Strain names are coloured according to their SGs. Asterisks indicate the genes disrupted by IS insertion or other mutations. The genomic regions used for the first PCR in the two-step PCR for Stx2a phage subtyping [19] are indicated.

There was remarkable genomic diversity between the Stx2a phages in the closed clade 8 genomes (Figs 3 and S6). Based on inspection of their genomic structures, although all Stx2a phages are short-tailed phages having highly conserved late genes similar to the Stx2a phages of O157:H7 strains Sakai [73] and EDL933 [74], the early region displays notable genomic diversity (Fig. 4), which is related to the presence of two different subtypes of Stx2a phages in this clade (γ and δ subtypes) [19]. *In silico* PCR analysis of these Stx2a phages (*n*=32) using a set of PCR primers for Stx2a phage subtyping by two-step PCR [19] indicated 13 and 14 phages as γ and δ subtypes, respectively. However, the remaining five Stx2a phages were not clearly subtyped because they were subtyped as the γ subtype in the first PCR but negative in the second PCR. As the *o* and *p* replication genes of these five Stx2a phages exhibit notable sequence variation (97.9 % identity to those of γ Stx2a phages) and their regulatory genes (*cI*, *cro*, *cII*, and *n*) are very divergent from those of γ Stx2a phages, we defined them as γ variant 1 (γ_v1).

Although IS-associated small structural variations were frequently observed, the sequences of Stx2a phages are highly conserved within each subtype except for one δ phage in strain F690 (Fig. 4, see Fig. S11 for the results of all-to-all dot plot analysis of each subtype). Importantly, all SG8_30 strains as well as one SG8_31A and one SG8_31B strain contain γ Stx2a phages; γ_v1 phages are present in SG8_31A and δ phages in SG8_31B and SG8_32. Subtyping of Stx2a phages in the draft genomes by the read mapping strategy (see Methods) confirmed this subtype distribution following the phylogeny of host strains, though Stx2a phages in six strains could not be subtyped [we sequenced the early region of one of the six untypeable phages (that of strain 105_162091 in SG8_30) by the long PCR-based approach [19] as described in Methods and found that the *o* and *p* replication genes exhibit 96.6 % identity to those of δ Stx2a phages]. Moreover, the SG8_31A and SG8_31B strains containing γ Stx2a phages branched off early from the other strains in these groups, respectively. These results and the timed phylogeny of strains (Fig. S2) indicate that the MRCA of clade 8 contained the Stx2a phage of the γ subtype and that subtype changes occurred in SG8_31A and SG8_31B. While the subtype change in SG8_31A (from γ to γ_v1) was induced by replacement of a part of the early region required for replication and its regulation, the subtype change in SG8_31B (from γ to δ) was most likely caused by replacement of the entire phage genome. Three SG8_30 strains also acquired α Stx2a phages, and the α Stx2a phage in the closed genome (strain 8_140198) was integrated into the *wrbA* locus (Fig. 2).

It is worth mentioning that all Stx2a phages in the closed genomes contained at least one IS insertion. Although not all insertions affect phage propagation, many of these Stx2a phages carry morphogenic genes [75] inactivated by IS insertion. Moreover, we found several frameshift mutations that inactivated morphogenic genes. As shown in Figs 4 and S12, one or more morphogenic genes were inactivated in all γ and γ_v1 phages, except for the γ phage in strain CEC13002 (SG8_31A) and the γ_v1 phage in strain 53_142304 (SG8_31A). Thus, 12 of the 13 γ Stx2a phages and 4 of the 5 γ_v1 phages are apparently unable to produce infectious phage particles. In contrast, among the 14 δ Stx2a phages, head or tail formation genes have been inactivated by IS insertion in 5 phages (Figs 4 and S12).

### Stx2 production levels of clade 8 strains

**Fig. 5.**
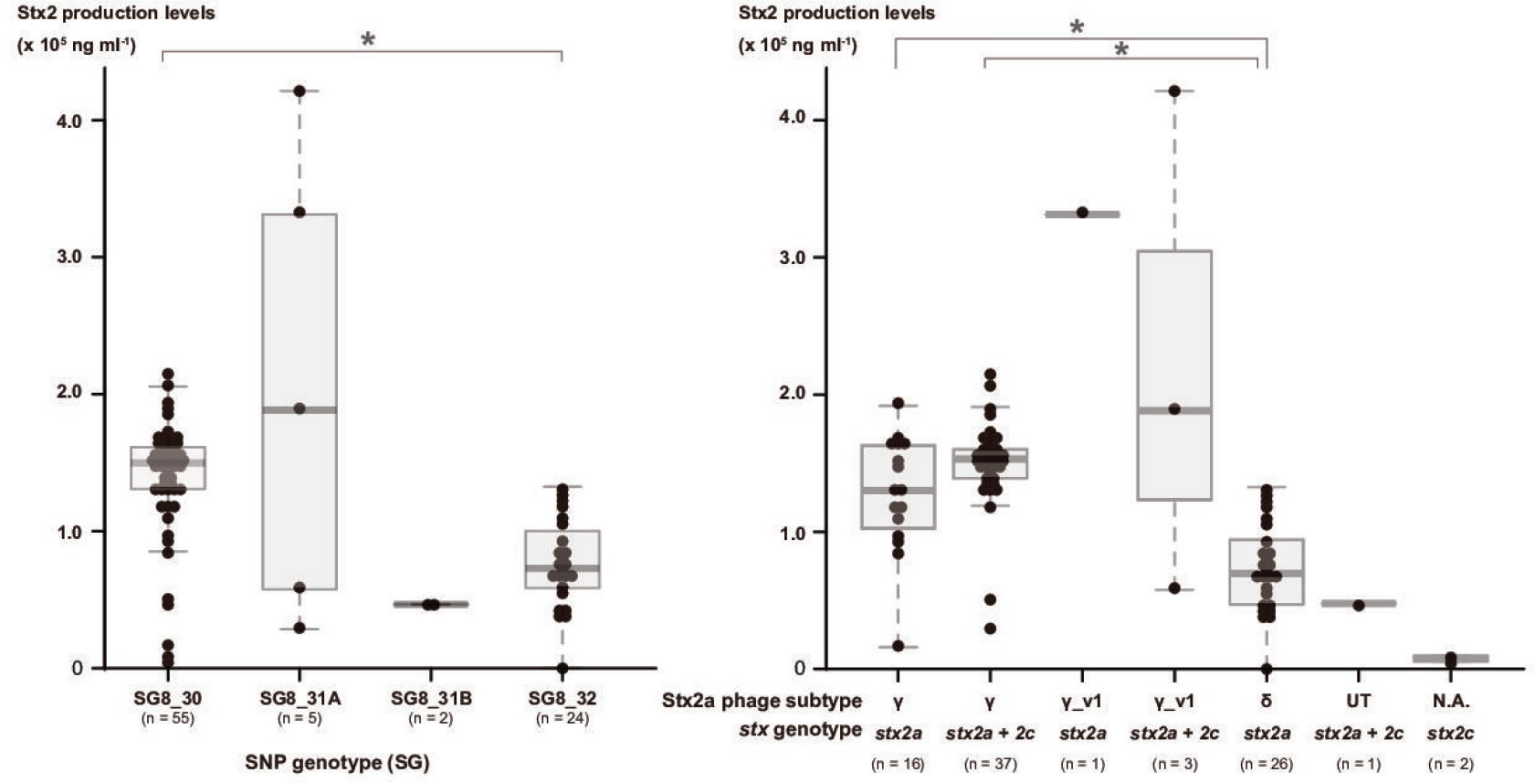
Comparison of the Stx2 production levels between SGs and between strains containing different Stx2a phage subtypes and *stx* genotypes. Statistical analysis was performed using the Kruskal‒Wallis test (with Bonferroni’s correction). Asterisks indicate *P* values of <0.05 (statistically significant). N.A.; not applicable

As three subtypes of Stx2a phages showed lineage-dependent distribution in clade 8, we re-evaluated the correlation of Stx2a phage subtypes (and host strain lineages) with the Stx2 production levels of host strains. To this end, we selected a total of 86 strains among those available in our laboratory, such that they covered the entire clade 8 population as much as possible; we then measured Stx2 production levels by FRET (fluorescence resonance energy transfer) [54] (Fig. 1). As depicted in Fig. 5, the mean production level of SG8_30 strains (all carrying γ Stx2a phages) was two times higher than that of SG8_32 strains (all carrying δ Stx2a phages) (1.5×10^5^ vs 7.3×10^4^ ng ml^−1^, *P* = <0.01). Although the SG8_31B strains we were able to examine were limited, two SG8_31B strains (both carrying δ Stx2a phages) showed low Stx2 production levels (4.6×10^4^ and 4.7×10^4^ ng ml^−1^). In addition, the mean Stx2 production level of strains carrying γ Stx2a phages was found to be significantly higher than that of strains carrying δ Stx2a phages, regardless of the presence or absence of *stx2c* [γ (*stx2a* alone) vs δ, 1.3×10^5^ vs 7.0×10^4^ ng ml^−1^ (*P*=0.0017); γ (*stx2a+stc2*c) vs δ, 1.5×10^5^ vs 7.0×10^4^ ng ml^−1^ (*P* = <0.001)]. The 86 strains included only 2 strains that contain *stx2c* alone, and their Stx2 production levels were very low (5.4×10^3^ and 9.9×10^3^ ng ml^−1^), as previously reported [19]. These results indicate that the presence of Stx2c phages does not affect the Stx2 production levels of clade 8 under the conditions used in this study. To further understand how the coexistence of *stx2a* and *stx2c* affects Stx2 production levels, we measured the Stx2 levels of subclones of two *stx2a*/*stx2c* strains (53_142304 and 57_142493), in which spontaneous deletion of Stx2a phage occurred. As expected, the Stx2 levels of subclones lacking *stx2a* were much lower than those of the parent strains (1.0×10^4^ vs 5.7×10^4^ ng ml^−1^ in strain 53_142304 and 5.1×10^3^ vs 1.9×10^5^ ng ml^−1^ in strain 57_142493).

Although SG8_31A strains we could examine were also limited (*n*=5), two showed very high levels of Stx2 production (strain CEC13004 3.3×10^5^ ng ml^−1^, strain 93_161312 4.2×10^5^ ng ml^−1^), and the Stx2 production level of one strain was also higher than the SG8_30 strain mean value (strain 57_142493 1.9×10^5^ ng ml^−1^). These three strains carry γ_v1 Stx2a_phages. In contrast, another γ_v1 phage-containing strain showed a relatively low production level similar to that of δ phage-containing strains (strain 53_142304 5.7×10^4^ ng ml^−1^). However, the *n* antiterminator gene has been inactivated by IS*1203* insertion (Fig. 4), and this mutation may affect the efficiency of phage induction and, therefore, expression of the *stx2a* gene.

### Correlation of lineages (SGs) and disease severity within clade 8

Taking advantage of the fact that the clinical information of 132 Japanese strains is available, we analysed difference in disease severity between different SGs within clade 8. Here, severe diseases are defined as positive for bloody diarrhoea or HUS. As shown in Table 3, patients infected by SG8_30 strains were more likely to develop severe disease [odds ratio (OR) 2.92, 95 % confidence interval (CI) 1.12–7.61, *P*=0.018], whereas patients infected by SG8_32 strains were less likely to develop severe disease (OR 0.32, 95 % CI 0.12–0.90, *P*=0.019). As SG lineages of clade 8 strains and their Stx2a phage subtypes correlated well, patients infected by γ Stx2a phage-carrying strains were more likely to develop severe disease (OR 3.21, 95 % CI 1.20–8.65, *P*=0.014), and patients infected by δ phage-carrying strains were less likely (OR 0.36, 95 % CI 0.13–1.02, *P*=0.041). Although the evaluation of SG8_31A and SG8_31B strains was difficult because such strains available for this study were very limited, these results indicate that infection by SG8_30 strains (all strains contain γ Stx2a phages and showed higher Stx2 levels) is associated with a higher risk of severe disease development among clade 8 infections; conversely, infection by SG8_32 strains (all contain δ Stx2a phages and showed lower Stx2 levels) is associated with a lower risk of severe disease.

**Table 3.** Comparison of disease severity between different SGs and between different Stx2a phage subtypes na, Not applicable

Strain category	Incidence rate			Severe disease	
	BD*	HUS†	Severe disease‡	OR [95 % CI]	*P* value§
**SNP genotypes**					
SG8_30	79/98 (80.6 %)	13/98 (13.3 %)	81/98 (82.7 %)	2.92 [1.12–7.61]	**0.018**
SG8_31A	2/5 (40.0 %)	2/5 (40.0 %)	3/5 (60.0 %)	0.42 [0.047–5.35]	0.32
SG8_31B	2/2 (100 %)	0/2 (0 %)	2/2 (100 %)	na	na
SG8_32	14/27 (51.9 %)	4/27 (14.8 %)	16/27 (59.3 %)	0.32 [0.12–0.90]	**0.019**
Total	97/132 (73.5 %)	19/132 (14.4 %)	102/132 (77.3 %)	–	–
**Stx2a phage subtypes**					
γ	73/90 (81.1 %)	13/90 (14.4 %)	75/90 (83.3 %)	3.21 [1.20–8.65]	**0.014**
γ_v1	1/4 (25.0 %)	2/4 (50.0 %)	2/4 (50.0 %)	0.28 [0.020–4.08]	0.22
δ	16/29 (55.2 %)	4/29 (13.8 %)	18/29 (62.1 %)	0.36 [0.13–1.02]	**0.041**
Total	90/123 (73.2 %)	19/123 (15.4 %)	95/123 (77.2 %)	–	–

*Positive for bloody diarrhoea (regardless of the presence or absence of HUS).

†Positive for HUS (regardless of the presence or absence of the record of bloody diarrhoea).

‡Positive for bloody diarrhoea or HUS.

§Bold characters indicate *P* values <0.05.

## Discussion

In this study, we performed a large-scale genome analysis of a global strain set of STEC O157:H7 clade 8, a highly pathogenic lineage of this most prevalent STEC serotype worldwide. The strain set comprised 510 strains, including 147 Japanese strains sequenced in this study (Table 1). Of the 147 Japanese strains, the genome sequences of 18 strains were closed (Table 2), making it possible to perform detailed clade-wide genome analyses by using a total of 35 closed genomes that cover the entire clade 8 lineage. The variation in Stx2 production level between clade 8 strains, which may be associated with disease severity, and the reported clinical symptoms were evaluated within the context of strain phylogeny and Stx2a phage subtype. Through these analyses, we obtained several valuable findings on this important lineage of O157:H7.

First, we revealed the global population structure of clade 8 comprised four lineages, which correspond well to the SGs proposed by Manning *et al.* [17]: SG8_30, SG8_31A, SG8_31B and SG8_32 (Fig. 1). Their phylogenetic relationships indicate that SG8_30 and the common ancestor of the other lineages first became separated in clade 8 with SG8_31A and SG8_31B emerging from the latter and SG8_32 emerging from SG8_31B. SG8_33 emerged from SG8_32, but as it represents a sublineage of SG8_32, SG8_33 is not a useful classification category. Although no strains corresponding to an SG called SG8_34 [17] were included in our strain set, such strains likely represent a very minor subpopulation in clade 8 or the SG8_34 discriminating SNP may be a false SNP.

Second, comparison of 35 closed genomes provided a detailed view of the genomic diversity and conservation in clade 8. The chromosomes are well conserved in gene synteny (Fig. S4), but large inversions centred on the replication terminus do not appear to have been rare events (observed in 25.7 % of the 35 strains). All but one of the inversions were mediated by prophages (Fig. 2). The genomes of pO157 are also well conserved, except for various deletions in several strains and an interesting amplification of a 20 kb segment in strain F690 (Fig. S9). IS elements were apparently involved in the generation of these structural variations.

Regarding variation in the PP and IE content in clade 8, Eppinger *et al.* [21] performed an investigation of 11 strains; however, all but one were assigned to SG8_30 in this study. Thus, the present study provides a more extended view on this issue. Among the 29 PP/IE integration sites identified, 18 (62 %) are occupied by PPs/IEs in most strains (≥31/35), and 3 are occupied in 15–24 strains. Tandem integration of two PPs/IEs was also detected at five sites. Overall, the PPs/IEs found at the same locus are well conserved (Fig. 3), despite various deletions and insertions (Figs S6, S7 and S8). Stx2c phages were among such conserved PPs, indicating that the Stx2c phage present in the MRCA of clade 8 was lost twice, from SG8_32 and a sublineage in SG8_31B. An important exception is Stx2a phages. Although they have all been integrated into the *argW* gene (Fig. 2), clear subtype changes occurred in clade 8 (Fig. 1). This result is consistent with our previous report showing the presence of two Stx2a phage subtypes (γ and δ) in clade 8 [19], but a variant of the γ subtype (γ_v1) was additionally identified in this study. The phylogenetic distribution of the three subtypes (including γ_v1) clearly indicates that the MRCA of clade 8 carries an Stx2a phage of the γ subtype, which is replaced by the γ_v1 subtype in SG8_31A and by the δ subtype in SG8_31B and SG8_32 (Fig. 1). The subtype change from γ to γ_v1 was apparently caused by replacement of a part of early region containing genes for replication and early gene regulation (Fig. 4); in contrast, that from γ to δ was caused by complete (or nearly complete) PP replacement (Fig. 4). A detailed intralineage genome comparison of Stx2a phages has also been conducted in STEC O121:H19, but their Stx2a phage genomes are highly conserved [41], showing a sharp contrast to the Stx2a phages of O157:H7 clade 8. It is also noteworthy that although each subtype of Stx2a phages has been stably maintained in each lineage in clade 8 (Fig. S11), at least one gene for morphogenesis has been inactivated in nearly all γ and γ_v1 Stx2a phages (Fig. S12). Because such gene inactivation was less frequently observed in δ Stx2a phages (Fig. S12), this may be a simple reflection of the long persistence of the γ (and γ_v1) Stx2a phage in clade 8 (Fig. S2) and may well explain why our repeated efforts to isolate γ Stx2a phages from clade 8 strains failed.

Third, this study provides results of a re-evaluation of variation in the Stx2 production level in clade 8. Regarding intraclade variation in the mitomycin C-induced Stx2 production, two reports have described conflicting results: (i) strains belonging to a subclade called clade 8a, which carries γ Stx2a phages and corresponds to SG8_30, produce more Stx2 than strains in the other subclade (clade 8b), which carries δ Stx2a phages and corresponds to SG8_31B and SG8_32 [19]; (ii) strains belonging to clade 8b produce more Stx2 than clade 8a strains [22]. The experimental protocols employed by the two studies differed in the media used (CAYE medium in the former and LB medium in the latter) and the preparation of samples (treated with polymyxin B in the former but not treated and, thus, Stx2 only in the culture supernatants was measured in the latter). It should also be mentioned that the clade 8 strains analysed in each study were collected in very limited geographical regions in Japan (Fukuoka city or Chiba prefecture). Moreover, the values reported by Hirai and colleagues [22] were those obtained by single measurement. In this study, under the same conditions, we determined the Stx2 production levels of 86 strains isolated in various regions in Japan, which phylogenetically represent the entire clade 8 population as much as possible and included the strains analysed in both of the above studies (Fig. 1). Our current analysis revealed that SG8_30 strains produce significantly higher levels of Stx2 than SG8_31B and SG8_32 strains. Thus, the Stx2 production levels of clade 8 strains correlated well with their phylogeny. As Stx2a phage subtypes also correlated with host strain phylogeny, Stx2 production levels correlated with Stx2a phage subtype. In addition, our analysis revealed the presence of two SG8_31A strains that harbour γ_v1 Stx2a phages and produced more Stx2 than SG8_30 strains, more than two times higher than the mean level of SG8_30 strains (Fig. 5). One γ_v1 Stx2a phage-carrying SG8_31A strain showed a low level of Stx2 production, but the *n* gene involved in the regulation of phage induction [76] was inactivated in this strain. Thus, there is a possibility that the Stx2 production levels of SG8_31A strains are generally higher than those of the other clade 8 strains, though more SG8_31A strains need to be analysed to confirm this hypothesis. It should also be noted that there is still no direct evidence to indicate that Stx2a phage subtype directly determines the Stx2 production level of host strains. One simple strategy to obtain such evidence is the generation of isogenic *E. coli* strains carrying each subtype of Stx2a phage, but as most sequenced γ and γ_v1 Stx2a phages are defective in infective virion formation, the use of γ and γ_v1 Stx2a phages that contain apparently intact morphogenic genes or a different approach will be needed. Regarding variation in Stx2 production level, it should also be noted that the presence of Stx2c phages does not affect the level, at least in strains carrying γ Stx2a phages (Fig. 5).

Finally, our analysis of the clinical records of 132 strains revealed a difference in the risk of severe disease development among clade 8 strains. When severe disease was defined as the onset of bloody diarrhoea and/or HUS, SG8_30 strains were linked to a higher risk than SG8_32 strains (Table 3). As SG8_30 strains produced a higher level of Stx2 (the production of which is known to be associated with severe disease [14–16]) than SG8_32 strains, this difference is potentially related to the difference in the risk of severe disease development. As no clear conclusion was obtained for SG8_31A and SG8_31B strains due to the small numbers available in this analysis, the risk of these strains should be evaluated in the future. Such analyses are particularly important for γ_v1 phage-containing SG8_31A strains because two of them produced a higher level of Stx2 than SG8_30 strains, and two of the four strains analysed (strain 93_161312 showing the highest level of Stx2 production and strain 53_142304 showing a modest level) were isolated from HUS patients. As mentioned above, it is not known whether SG8_31A strains generally produce higher levels of Stx2 than other clade 8 strains and to what extent the production levels of Stx2 observed *in vitro* correlate with those in the intestine of patients, but attention should be given to γ_v1 phage-containing SG8_31A, as these strains may have a higher potential to cause severe disease.

## Supplementary Data

Supplementary material 1

Supplementary material 2
