# Supplementary material for: Alteration of a Shiga toxin-encoding phage associated with a change in toxin production level and disease severity in Escherichia coli
Source: Microb Genom. 2023 Feb 23;9(2):mgen000935. doi: 10.1099/mgen.0.000935 (PMC9997748; doi:10.1099/mgen.0.000935)
Supplement: Supplementary material 1 [file mgen-9-935-s001.pdf]

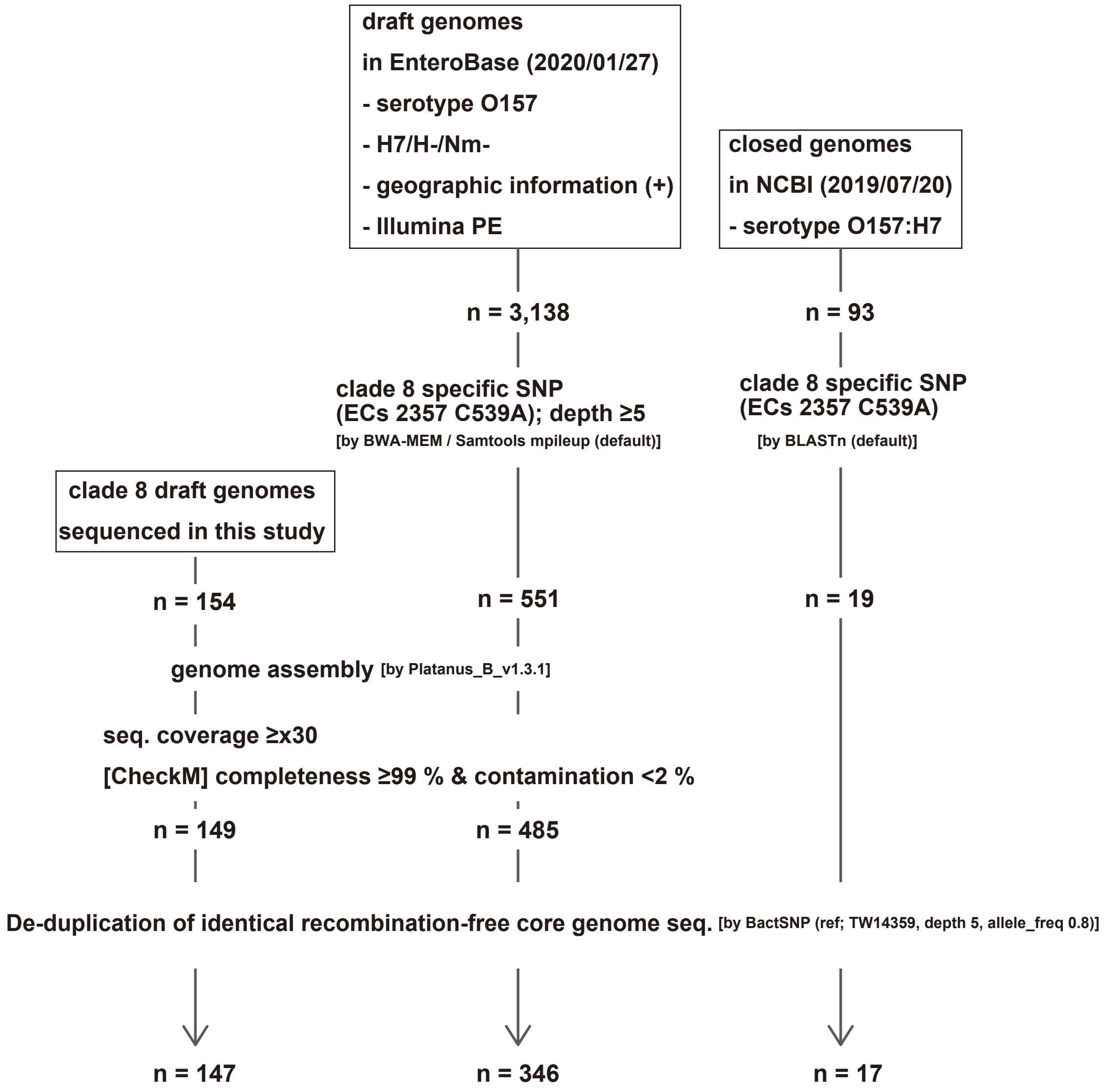

Fig. S1. The process and thresholds for the selection of clade 8 strains analyzed in this study. Completeness and contamination were assessed by CheckM [Parks DH, Imelfort M, Skennerton CT, Hugenholtz P, Tyson GW. CheckM: assessing the quality of microbial genomes recovered from isolates, single cells, and metagenomes. *Genome Res* 2015;25(7):1043-1055].

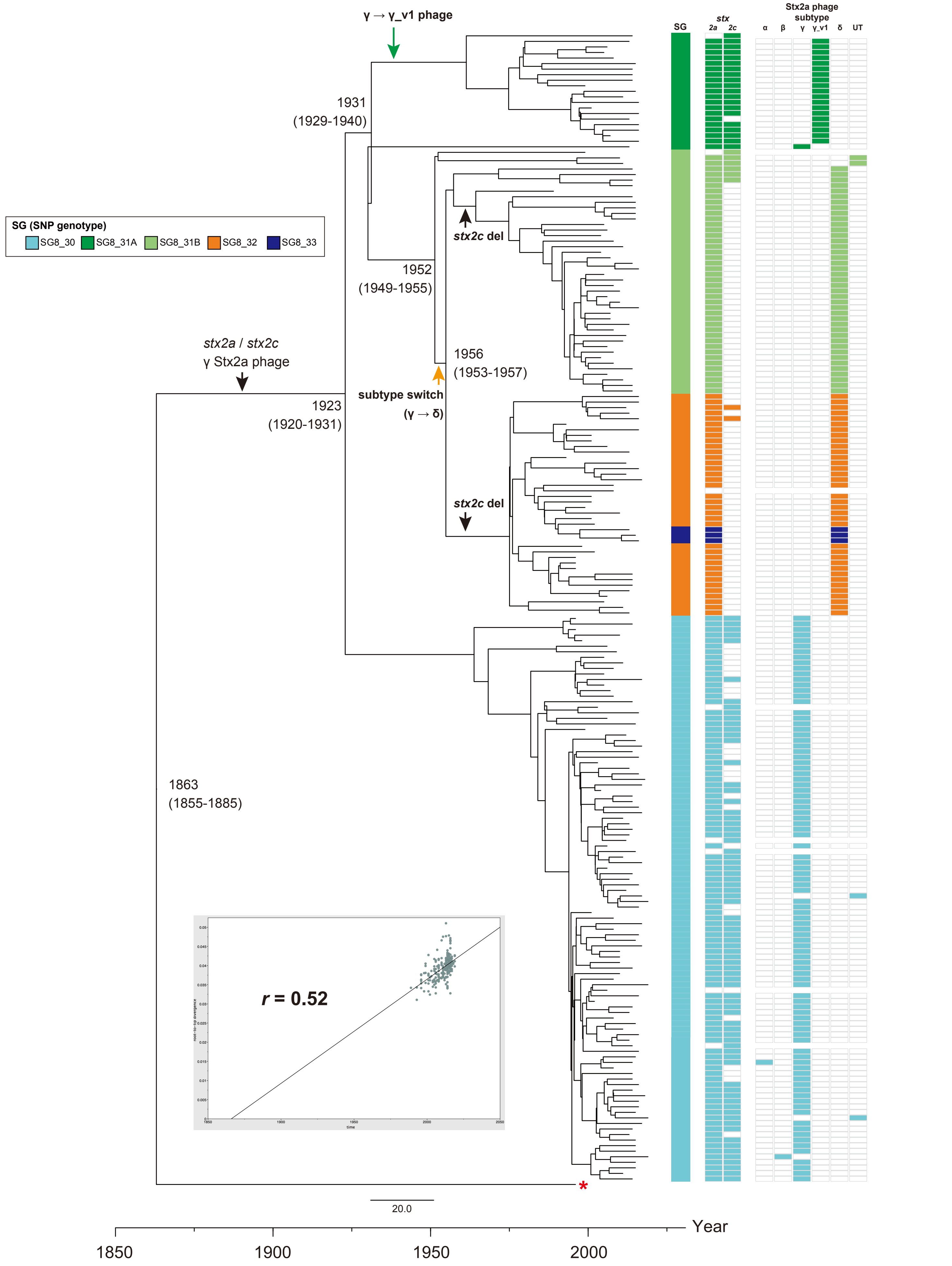

Fig. S2. A maximum clade credibility tree representing the evolutionary history of O157:H7 clade 8 strains (n=207) inferred by Bayesian evolutionary analysis. The 207 clade 8 strains analysed were selected by Treemmer (see Methods in the main text). The result of regression analysis of root-to-tip distance against sampling date is shown in the inset. The time to the most recent common ancestor of each clade 8 lineage is shown in the tree with the 95% highest posterior density (HPD) indicated in parentheses. SNP genotypes (SG), *stx* genotypes, and Stx2a phage subtypes are shown and colored according to the SG of each strain. Evolutionary events of *stx2* gain/loss and Stx2a phage subtype switch are indicated in the tree. *E. coli* O157:H7 strain Sakai [clade 1] was used as an outgroup (red asterisk).

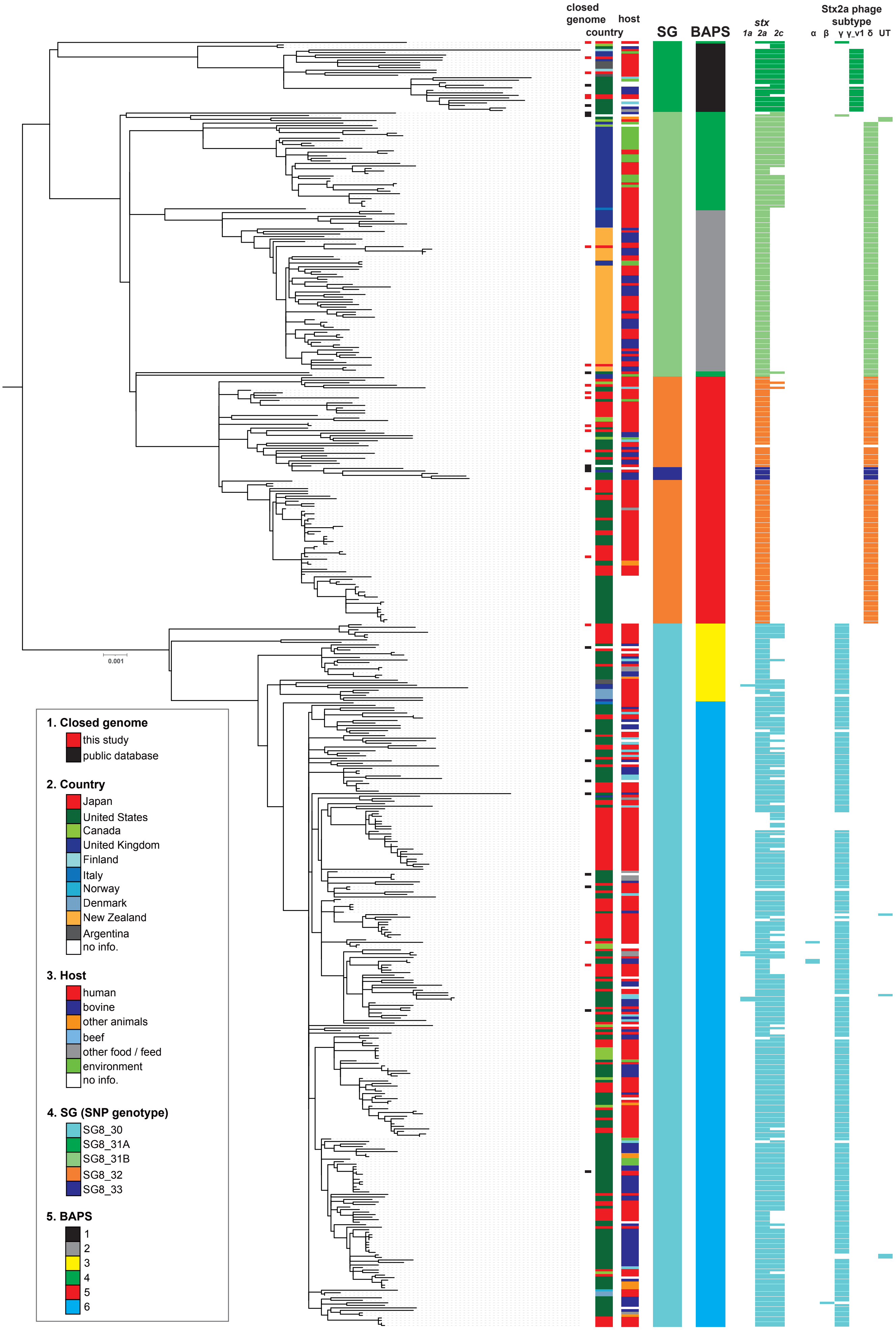

Fig. S3. Genomic clusters inferred by Bayesian analysis of population structure (BAPS). The ML tree and strain information (closed genome, country, host, SNP genotype (SG) and *stx* genotype, and Stx2a phage subtype) are the same as those in Fig. 1 in the main text.

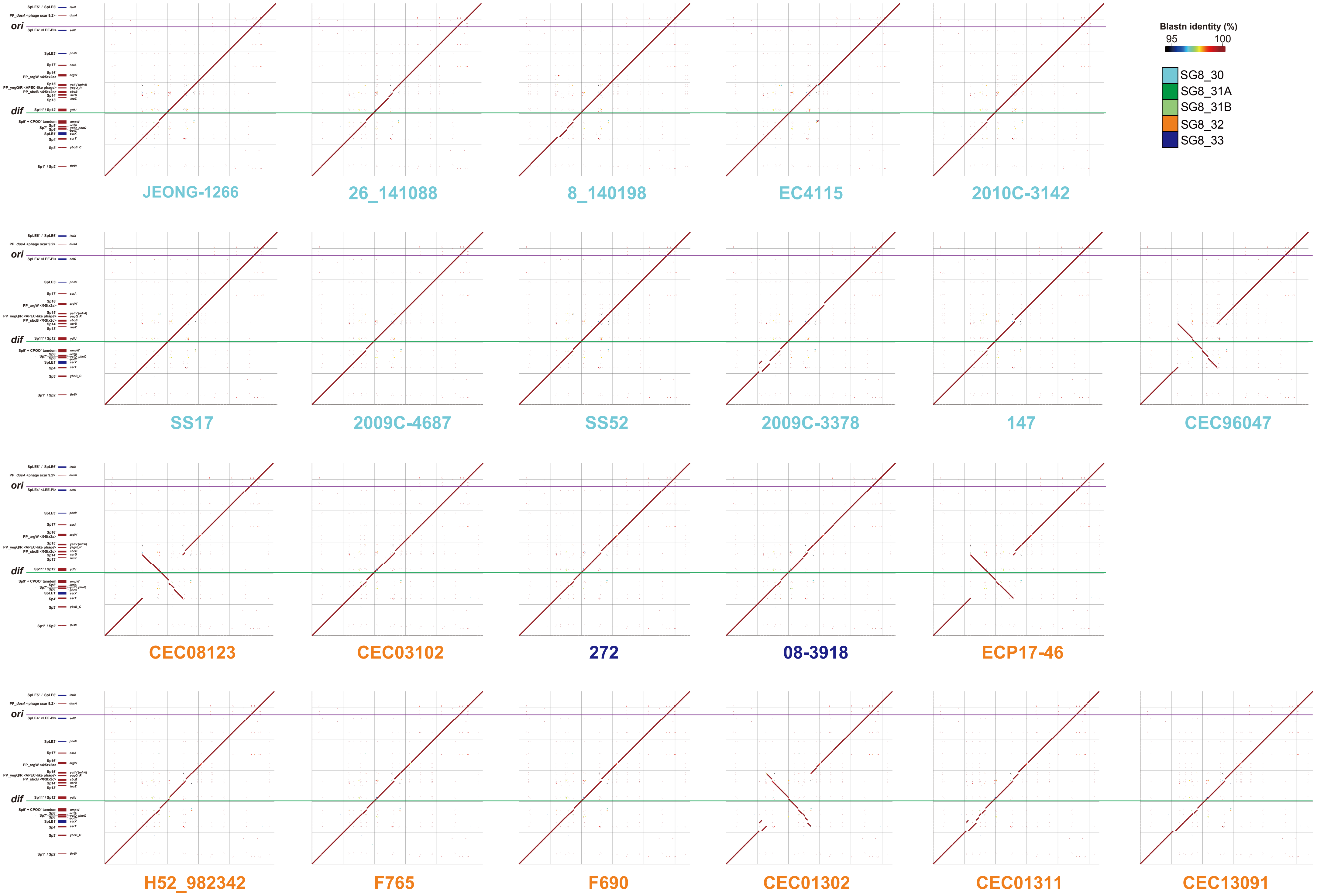

SpLE5' / SpLE6'
leuX
PP_dusA <phage scar 9.2>
dusA
ori
SpLE4' <LEE-PI>
selC
SpLE3'
pheV
Sp17'
ssrA
Sp16'
PP_argW <ΦStx2a>
argW
Sp15'
yehV (mlrA)
PP_yegQ/R <APEC-like phage>
yegQ_R
PP_sbcB <ΦStx2c>
sbcB
Sp14'
serU
Sp13'
leuZ
dif
Sp11' / Sp12'
ydfJ
Sp9' + CPOO' tamdem
ompW
Sp8'
icdA
Sp7'
ycfD_phoQ
Sp6'
potC
SpLE1'
serX
Sp4'
serT
Sp3'
ybcB_C
Sp1' / Sp2'
thrW
Blastn identity (%)
95
100
SG8_30
SG8_31A
SG8_31B
SG8_32
SG8_33
JEONG-1266
26_141088
8_140198
EC4115
2010C-3142
SS17
2009C-4687
SS52
2009C-3378
147
CEC96047
CEC08123
CEC03102
272
08-3918
ECP17-46
H52_982342
F765
F690
CEC01302
CEC01311
CEC13091

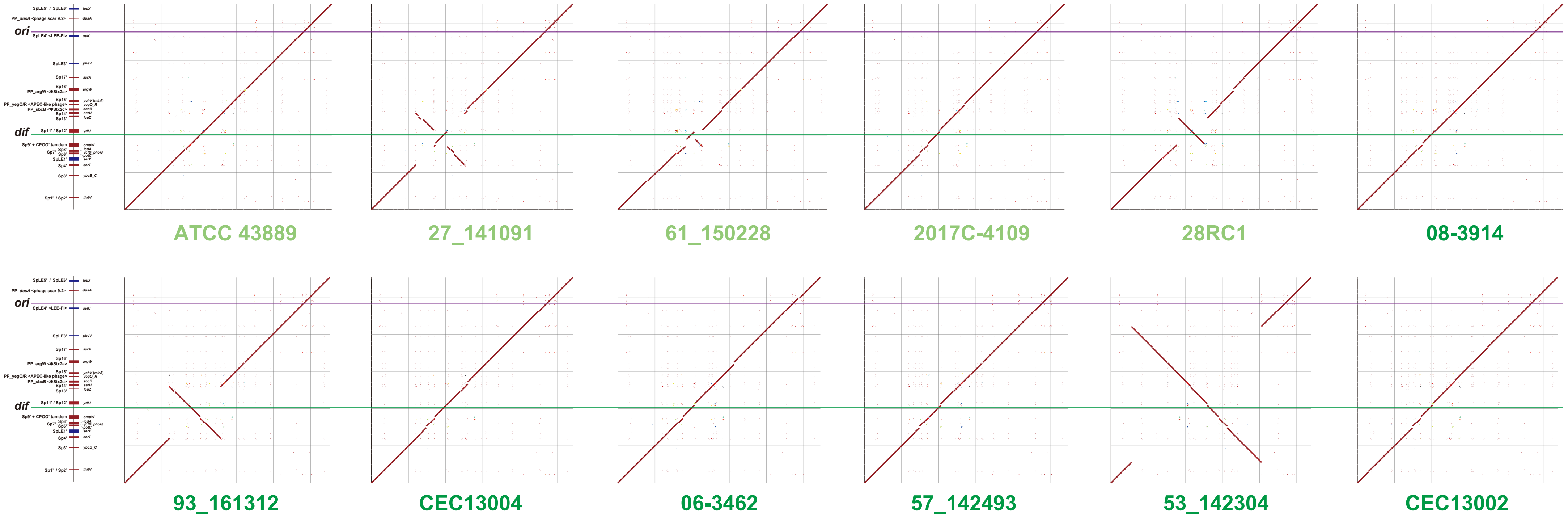

Fig. S4. Dot plot analyses of the chromosome sequences of 35 closed genomes. The chromosomes of 34 strains (x-axis) were compared with that of strain TW14359 (SG8_30) as a reference (y-axis). Strain names are colored according to their SGs. The rectangles on the y-axis indicate prophages (PPs; red) and integrative elements (IEs; blue) in strain TW14359. The positions of the origin (*ori*) and terminus (*dif*) of replication are also indicated.

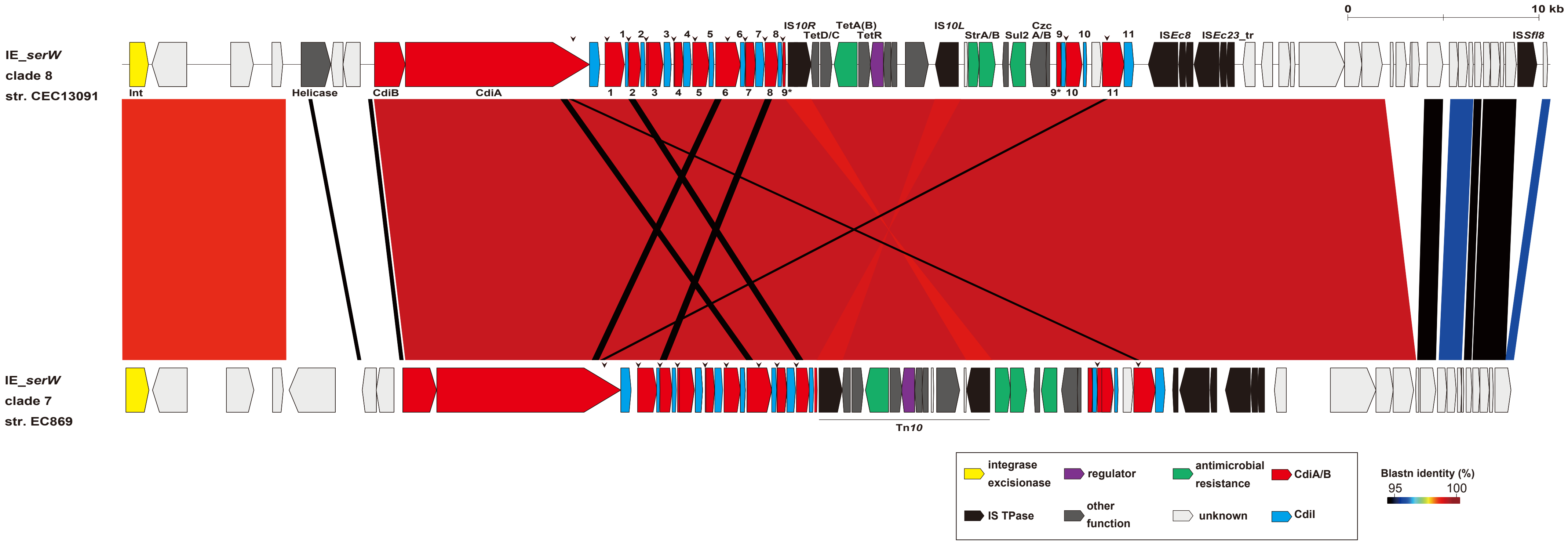

Fig. S5. The IE found at the *serW* locus in strain CEC13091 (SG8_32). A very similar element found in the *E. coli* O157:H7 strain EC869 (clade 7) reported by Eppinger *et al.* [Ref. 68 in the main text] is also shown (Accession no.; ABHU01000020). Eppinger *et al*. described the element as a P4-like element integrated into the *clpA* locus. However, we found that the element is integrated into the *serW* locus with an 18-bp target site duplication similar to the IE in strain CEC13091. Both IEs carry the SSuT element [Ref. 67 in the main text] encoding streptomycin, sulfonamide, and tetracycline resistance genes (the segment containing the tetracycline resistance gene corresponds to Tn*10*) and multiple genes for contact-dependent inhibition of growth (*cdi*). The *cdi* genes include three genes, each encoding full-length CdiB, CdiA, and CdiI (first three genes), and multiple pairs of genes encoding truncated CdiA/CdiI modules [Ref. 67 in the main text]. Black arrows indicate the VENN peptide motif at the start of truncated CdiA. The ninth CdiA/CdiI module is disrupted by insertion of the SSuT element.

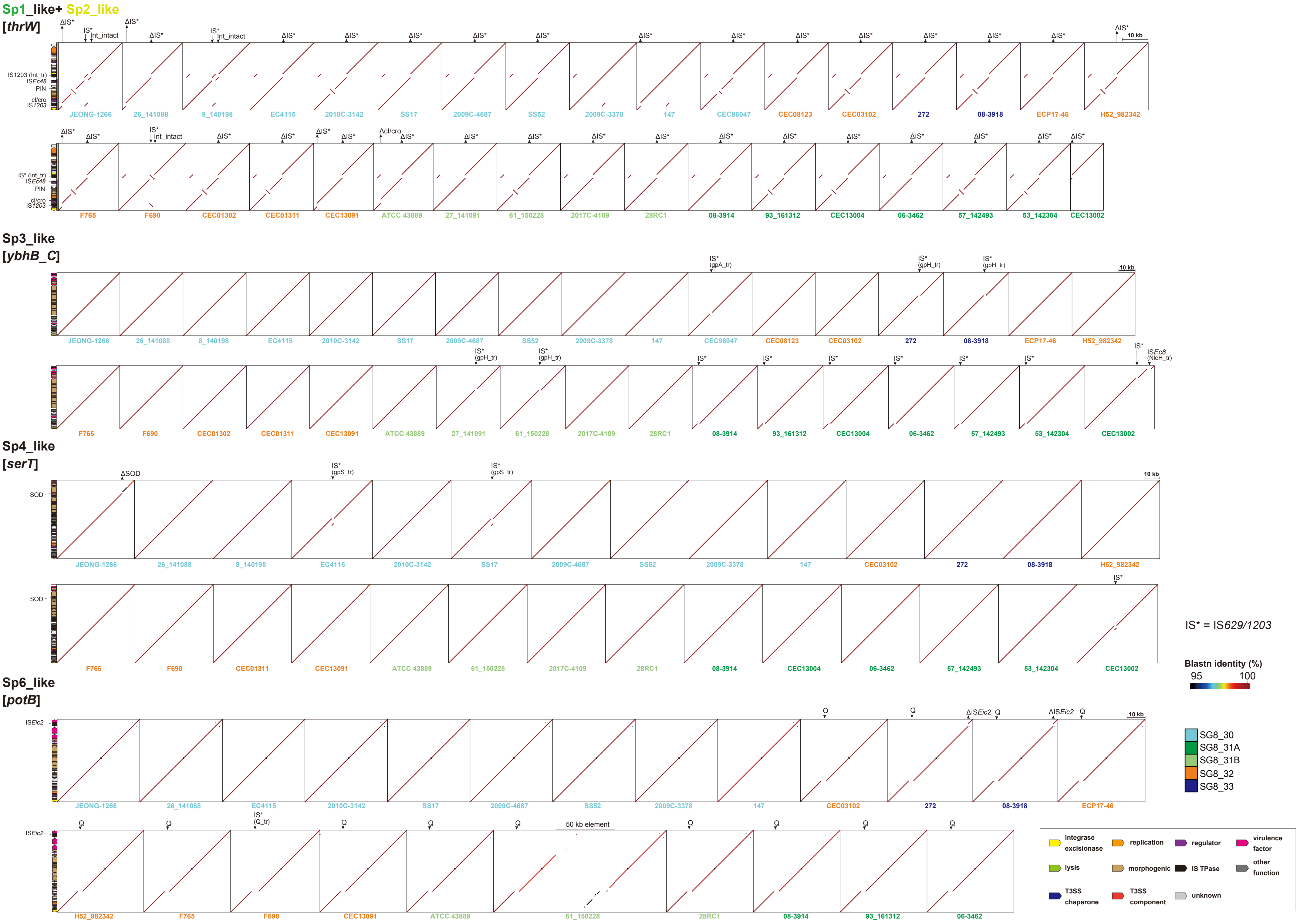

Sp1_like+ Sp2_like
[thrW]
Sp3_like
[ybhB_C]
Sp4_like
[serT]
Sp6_like
[potB]
IS* = IS629/1203
Blastn identity (%)
95
100
SG8_30
SG8_31A
SG8_31B
SG8_32
SG8_33
integrase excisionase
replication
regulator
virulence factor
lysis
morphogenic
IS TPase
other function
T3SS chaperone
T3SS component
unknown
50 kb element
10 kb

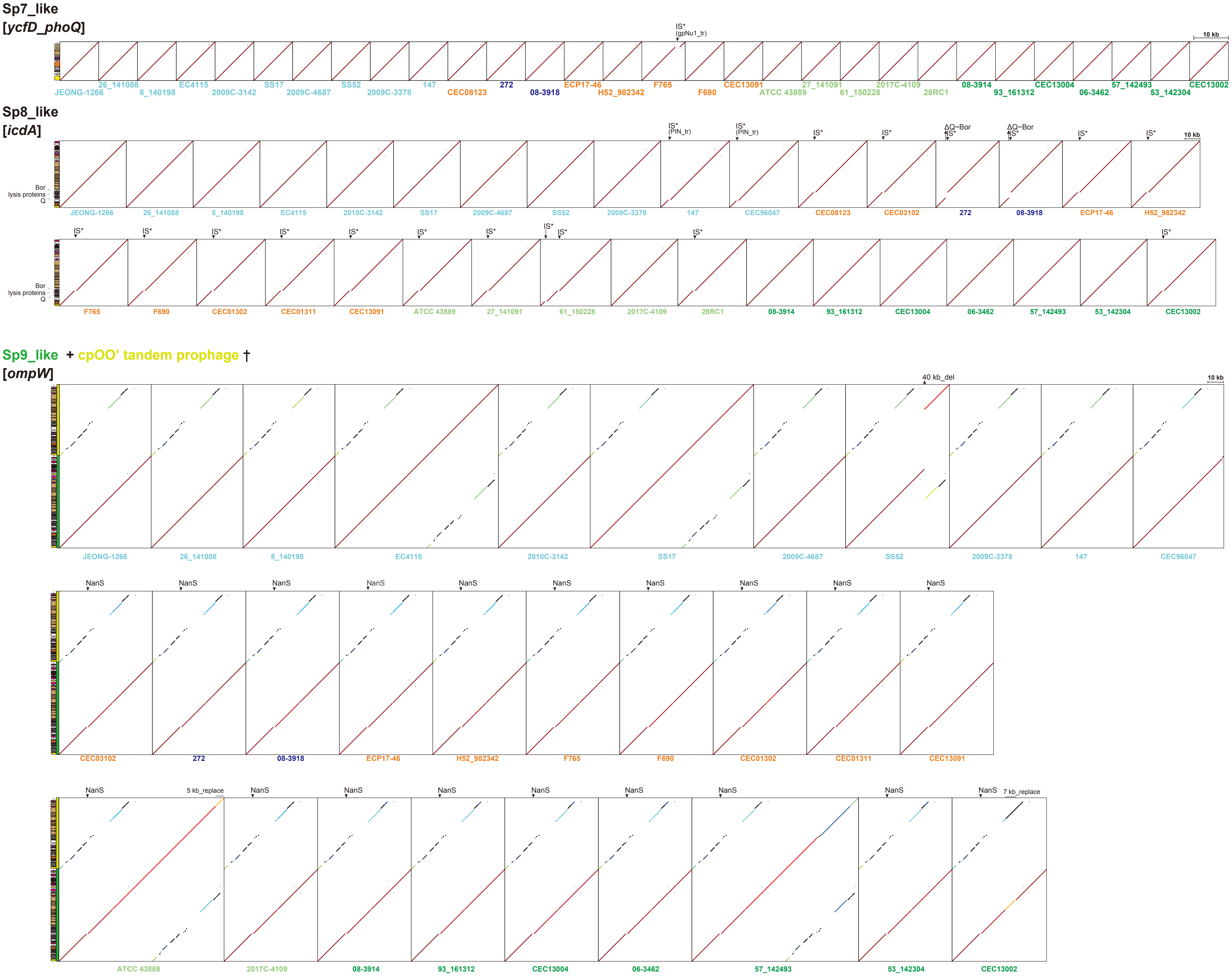
Sp7_like
[ycfD_phoQ]
IS*
(gpNu1_tr)
10 kb
JEONG-1266
26_141088
8_140198
EC4115
2009C-3142
SS17
2009C-4687
SS52
2009C-3378
147
CEC08123
272
08-3918
ECP17-46
H52_982342
F765
F690
CEC13091
ATCC 43889
27_141091
61_150228
2017C-4109
28RC1
08-3914
93_161312
CEC13004
06-3462
57_142493
53_142304
CEC13002
Sp8_like
[icdA]
Bor
lysis proteins
Q
IS*
(PIN_tr)
ΔQ~Bor
10 kb
JEONG-1266
26_141088
8_140198
EC4115
2010C-3142
SS17
2009C-4687
SS52
2009C-3378
147
CEC96047
CEC08123
CEC03102
272
08-3918
ECP17-46
H52_982342
F765
F690
CEC01302
CEC01311
CEC13091
ATCC 43889
27_141091
61_150228
2017C-4109
28RC1
08-3914
93_161312
CEC13004
06-3462
57_142493
53_142304
CEC13002
Sp9_like + cpOO' tandem prophage †
[ompW]
40 kb_del
10 kb
JEONG-1266
26_141088
8_140198
EC4115
2010C-3142
SS17
2009C-4687
SS52
2009C-3378
147
CEC96047
NanS
CEC03102
272
08-3918
ECP17-46
H52_982342
F765
F690
CEC01302
CEC01311
CEC13091
5 kb_replace
7 kb_replace
ATCC 43889
2017C-4109
08-3914
93_161312
CEC13004
06-3462
57_142493
53_142304
CEC13002

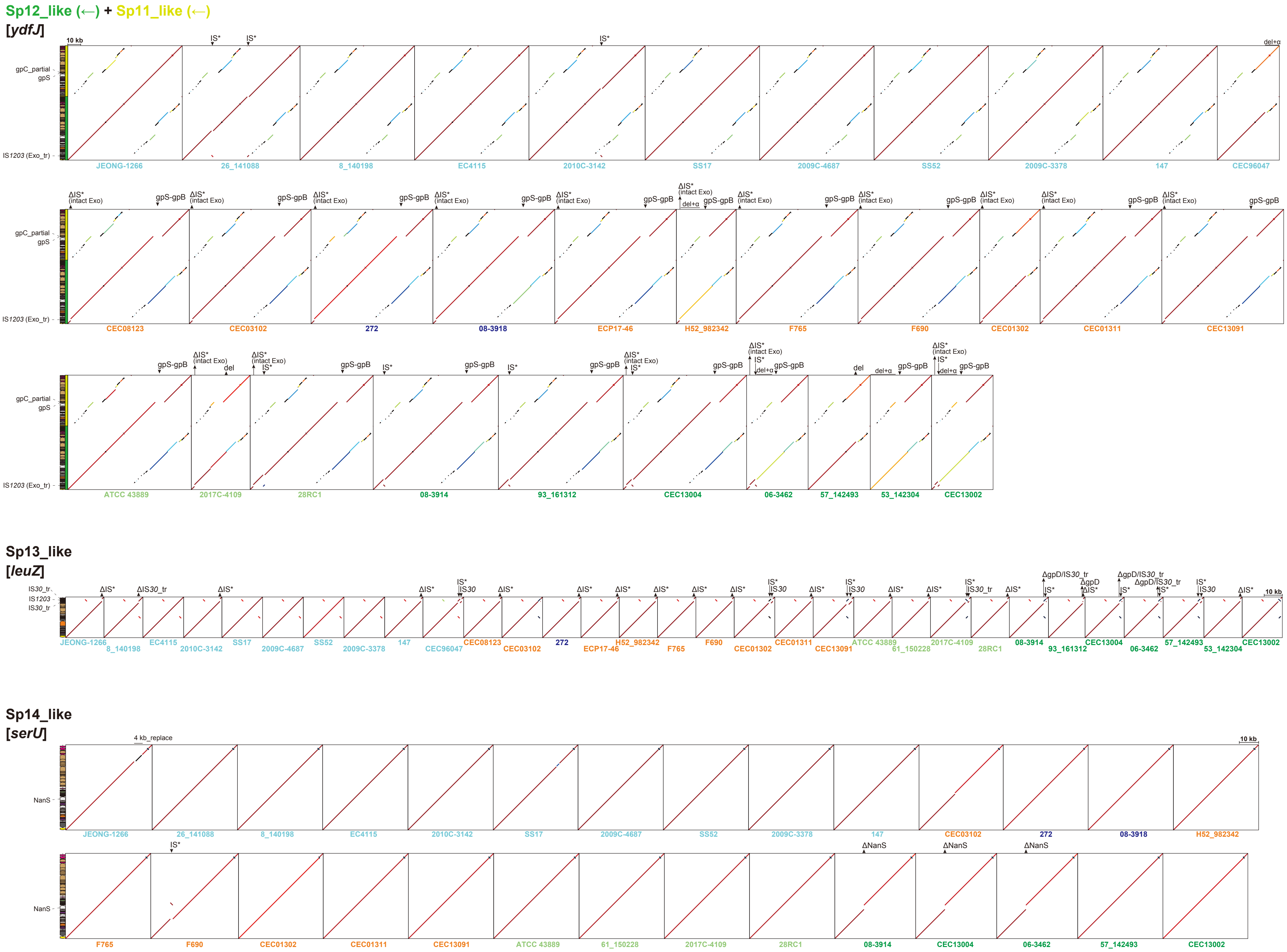
Sp12_like (←) + Sp11_like (←)
[ydfJ]
10 kb
gpC_partial
gpS
IS1203 (Exo_tr)
IS*
del+α
JEONG-1266
26_141088
8_140198
EC4115
2010C-3142
SS17
2009C-4687
SS52
2009C-3378
147
CEC96047
ΔIS* (intact Exo)
gpS-gpB
CEC08123
CEC03102
272
08-3918
ECP17-46
H52_982342
F765
F690
CEC01302
CEC01311
CEC13091
del
ATCC 43889
2017C-4109
28RC1
08-3914
93_161312
CEC13004
06-3462
57_142493
53_142304
CEC13002
Sp13_like
[leuZ]
IS30_tr
IS1203
ΔIS*
ΔIS30_tr
IS30
ΔgpD/IS30_tr
ΔgpD
61_150228
Sp14_like
[serU]
4 kb_replace
NanS
ΔNanS

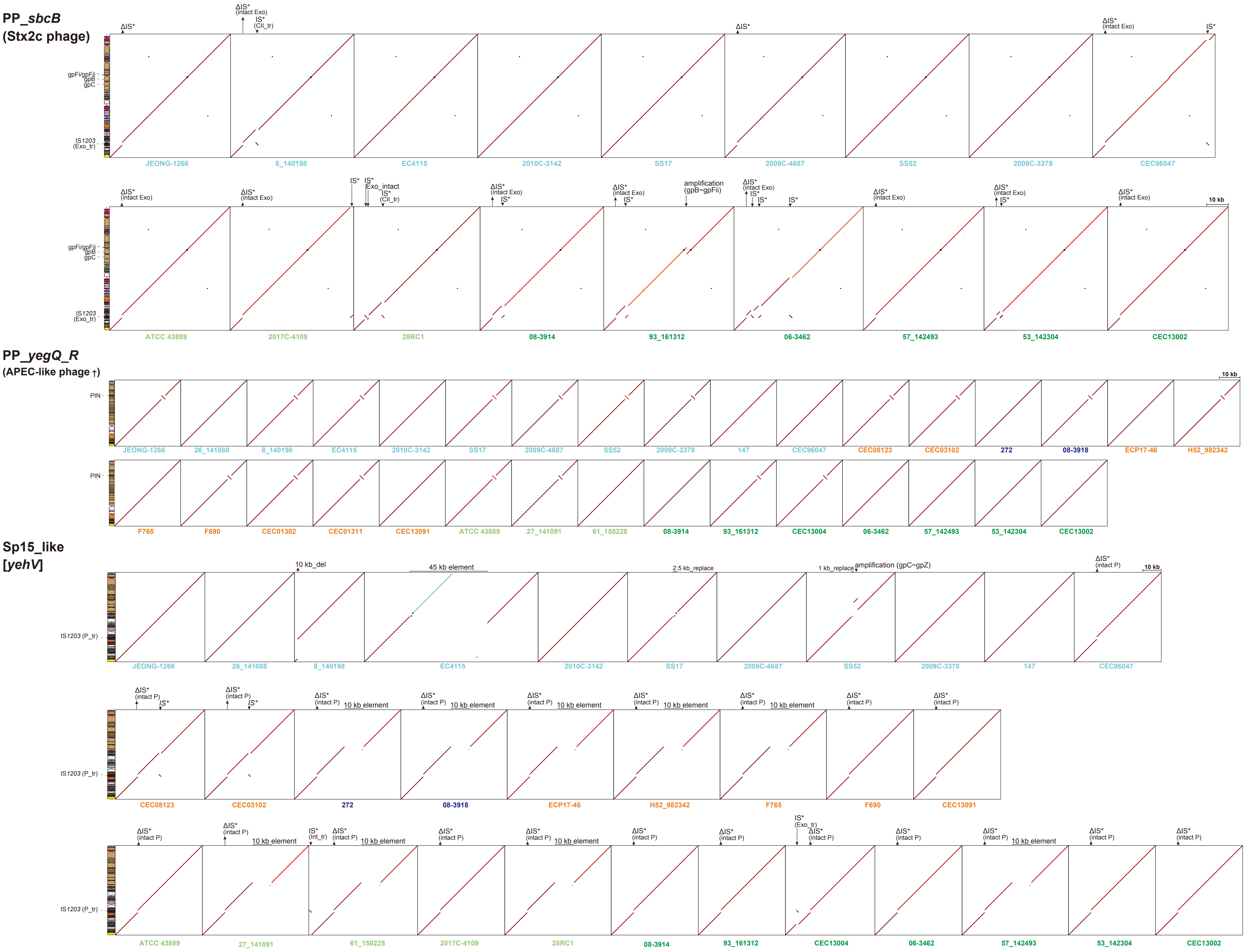
PP_sbcB
(Stx2c phage)
gpFi/gpFii
gpB
gpC
IS1203
(Exo_tr)
ΔIS*
(intact Exo)
IS*
(CII_tr)
JEONG-1266
8_140198
EC4115
2010C-3142
SS17
2009C-4687
SS52
2009C-3378
CEC96047
Exo_intact
amplification
(gpB~gpFii)
10 kb
ATCC 43889
2017C-4109
28RC1
08-3914
93_161312
06-3462
57_142493
53_142304
CEC13002
PP_yegQ_R
(APEC-like phage †)
PIN
26_141088
147
CEC08123
CEC03102
272
08-3918
ECP17-46
H52_982342
F765
F690
CEC01302
CEC01311
CEC13091
27_141091
61_150228
CEC13004
Sp15_like
[yehV]
IS1203 (P_tr)
10 kb_del
45 kb element
2.5 kb_replace
1 kb_replace
amplification (gpC~gpZ)
(intact P)
10 kb element
(Int_tr)
(Exo_tr)

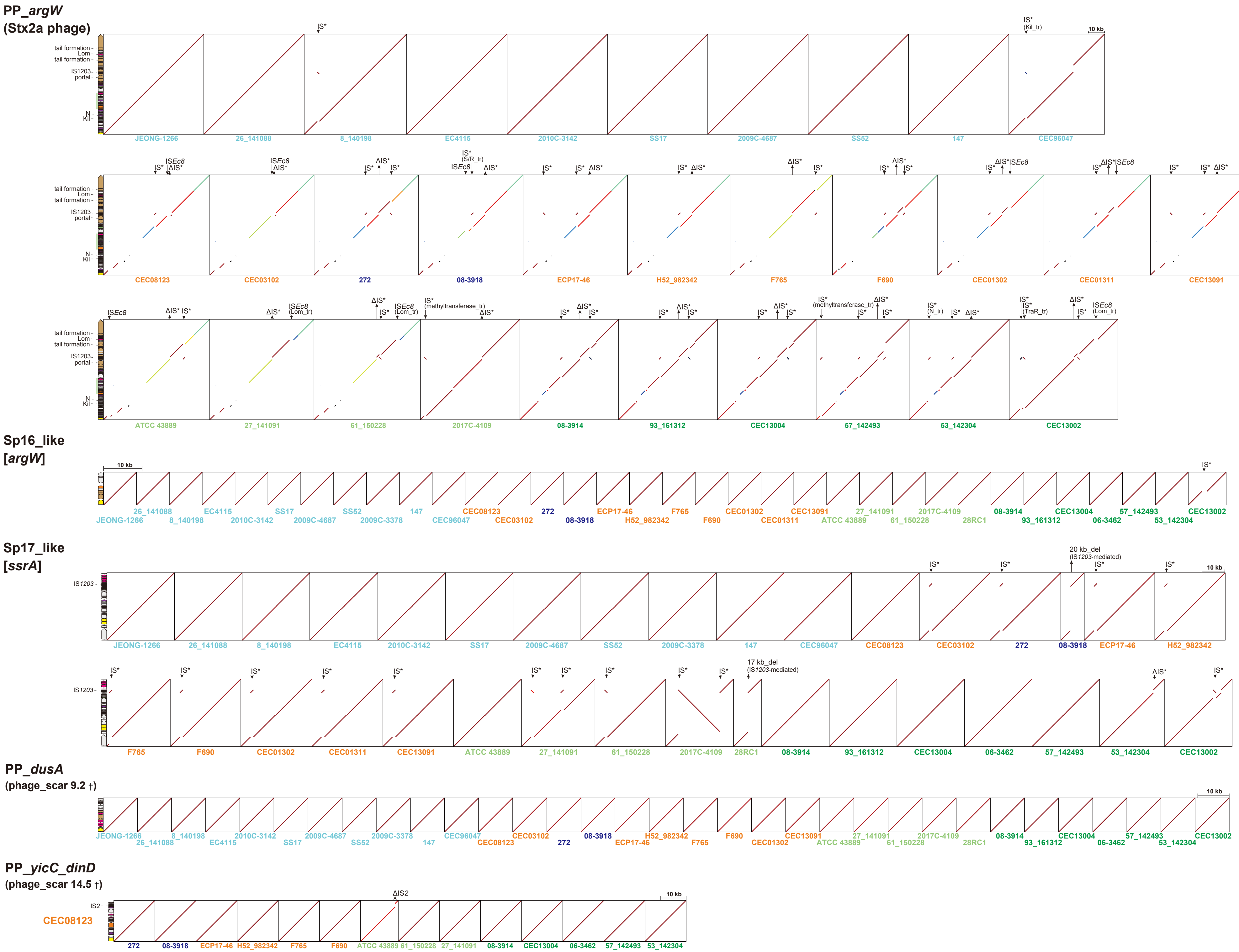
PP_argW
(Stx2a phage)
Sp16_like
[argW]
Sp17_like
[ssrA]
PP_dusA
(phage_scar 9.2 †)
PP_yicC_dinD
(phage_scar 14.5 †)
CEC08123
10 kb
tail formation
Lom
IS1203
portal
N
Kil
IS*
ISEc8
ΔIS*
ΔIS2
20 kb_del
(IS1203-mediated)
17 kb_del
(IS1203-mediated)
JEONG-1266
26_141088
8_140198
EC4115
2010C-3142
SS17
2009C-4687
SS52
147
CEC96047
CEC03102
272
08-3918
ECP17-46
H52_982342
F765
F690
CEC01302
CEC01311
CEC13091
ATCC 43889
27_141091
61_150228
2017C-4109
08-3914
93_161312
CEC13004
57_142493
53_142304
CEC13002
2009C-3378
28RC1
06-3462

SpLE1_like
[*serX*]

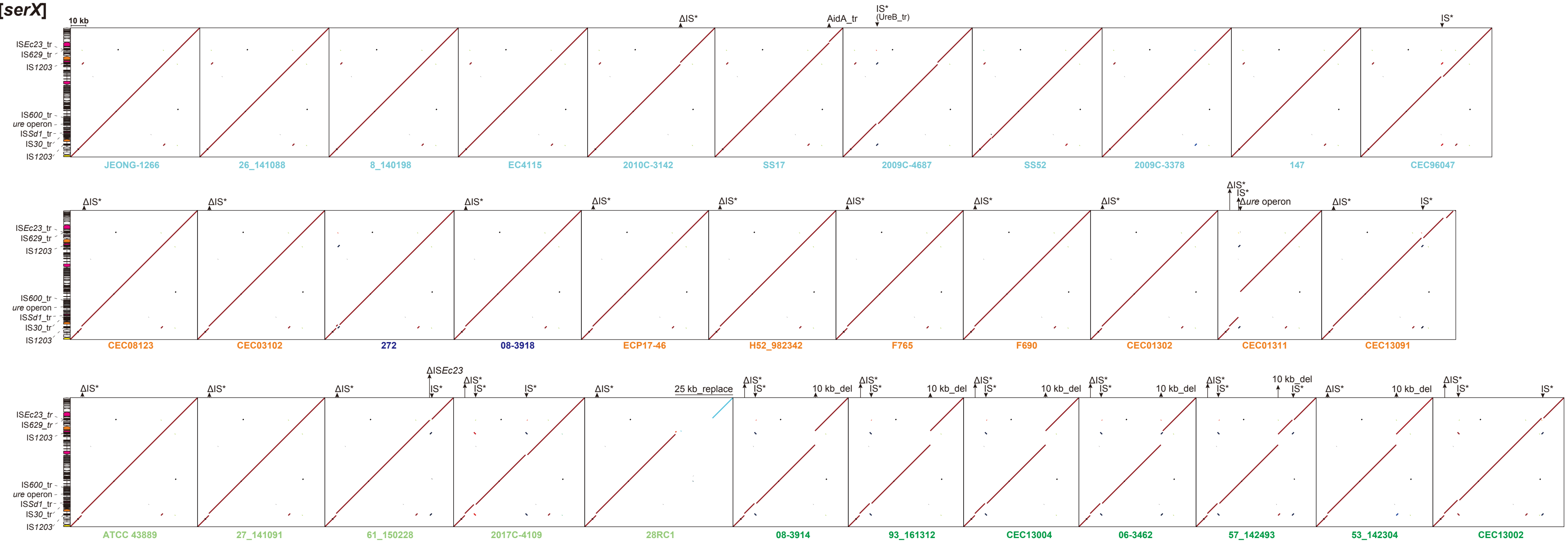

SpLE3_like
[*pheV*]

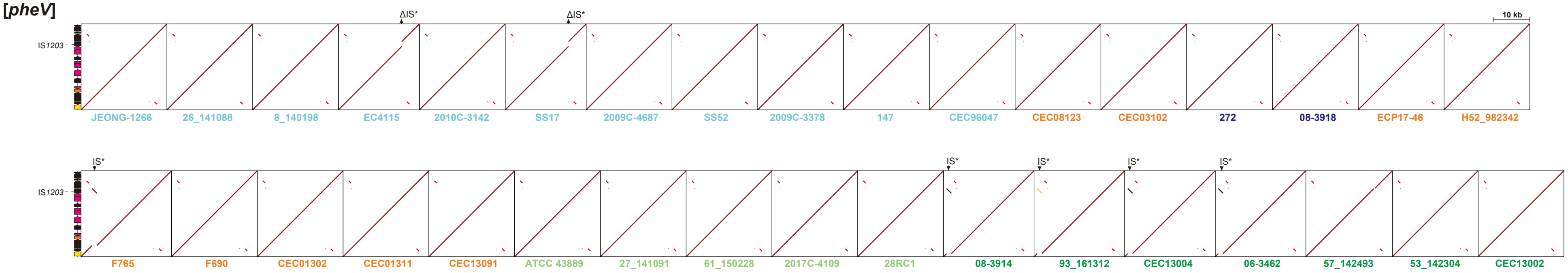

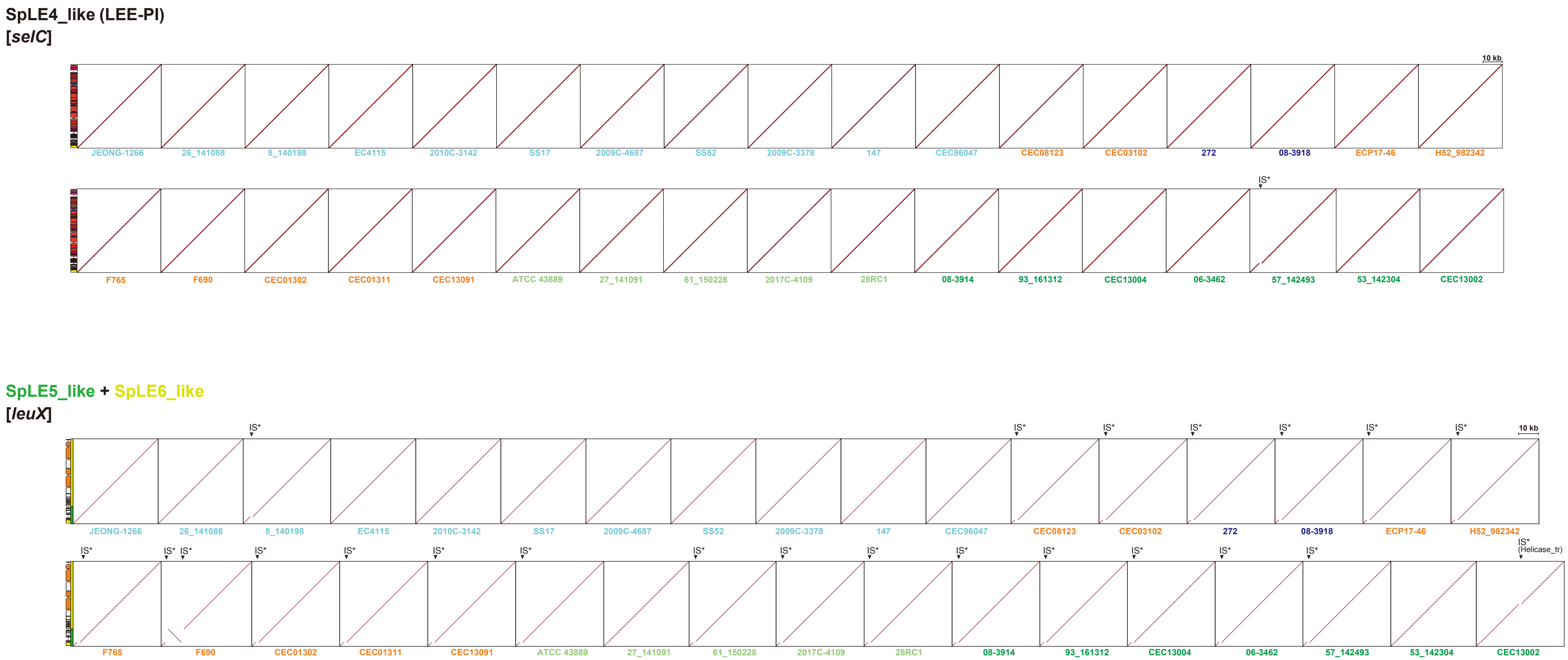

Fig. S6. Dot plot analyses of the PPs and IEs integrated into the same locus. The sequences of PPs/IEs in strain TW14359 (SG 8_30) were used as references (y-axis), except that the PP in strain CEC08123 was used as a reference for the PP integrated at the *yicC_dinD* locus because strain TW14359 does not contain this PP. Strain names are colored according to their SGs, and the PP names indicated by daggers are the names used by Eppinger *et al.* [Ref. 21 in the main text].

Lambda-like phages

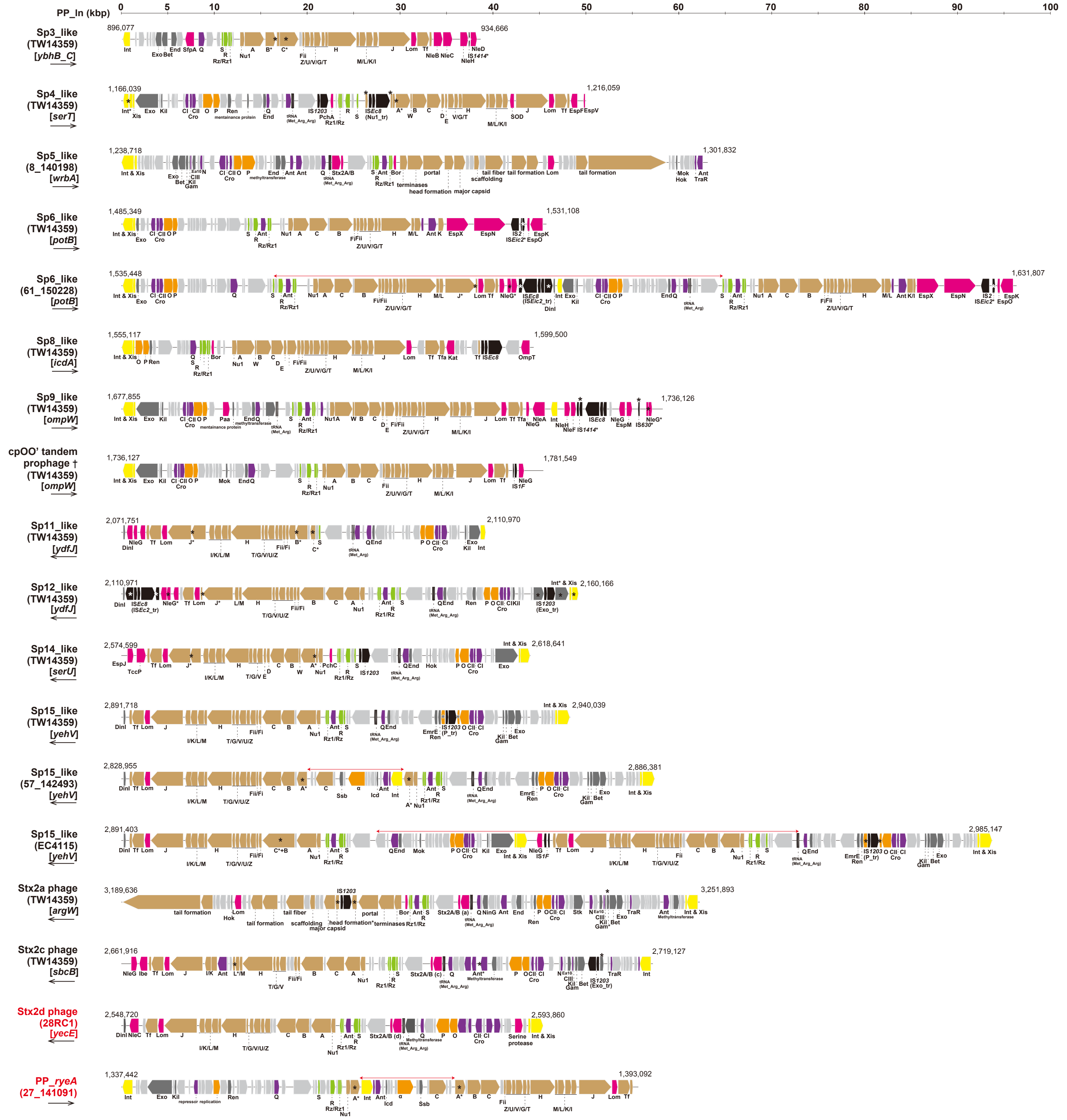

P2-like phages

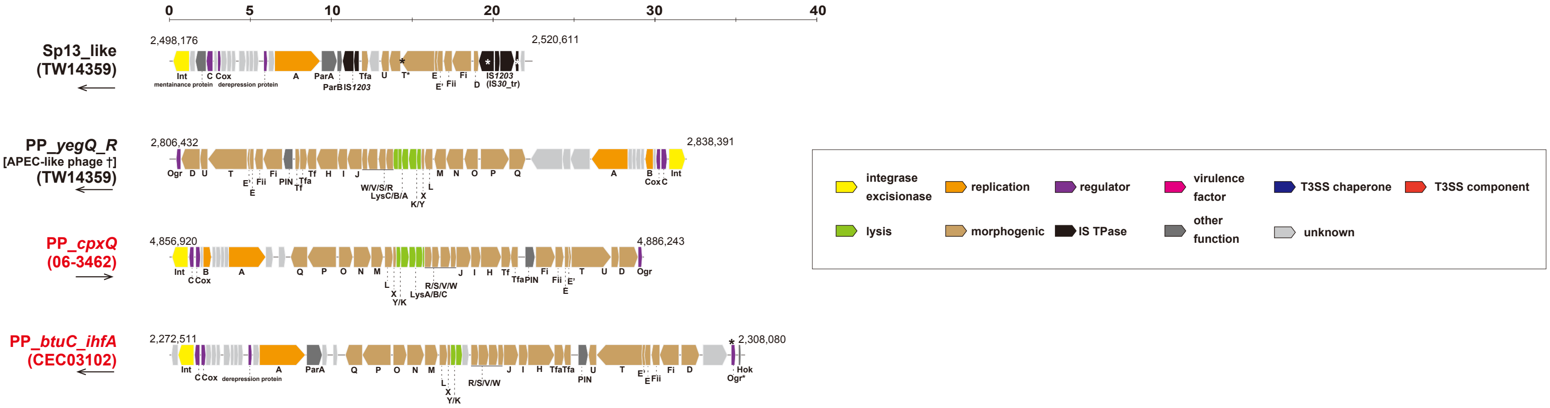

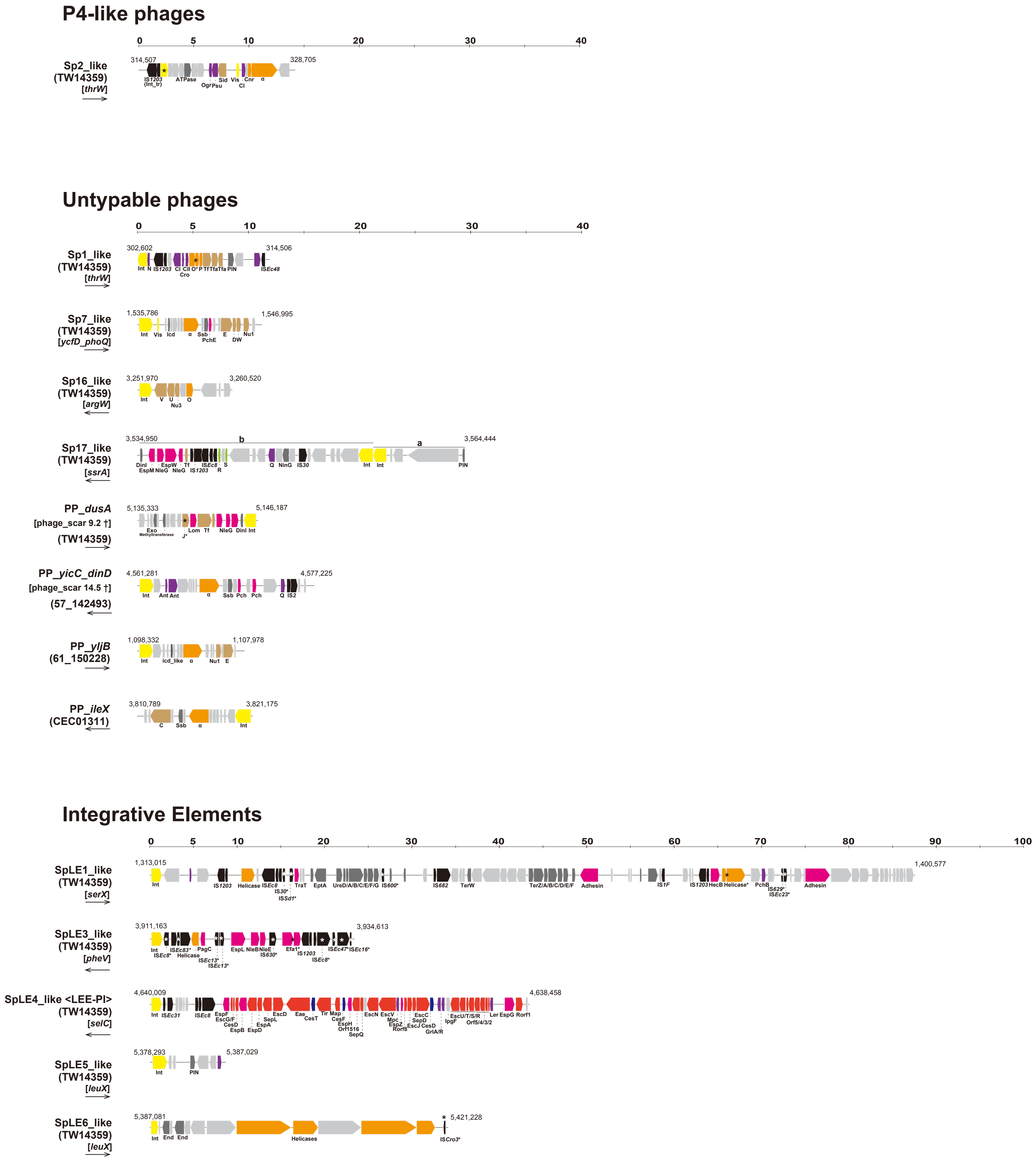

Fig. S7. Genomic structures of PPs and IEs identified in 35 closed clade 8 genomes. As PPs/IEs found at the same integration site are often very similar in sequence, representative PPs/IEs (mainly those found in strain TW14359 but also those in other clade 8 strains if there are considerable variations) are shown. The integration sites and positions of PPs/IEs in host chromosomes are also indicated. The direction of arrows indicates the direction of major transcripts relative to the direction of chromosome replication. Daggers indicate the PP name used by Eppinger *et al.* [Ref. 21 in the main text]. The IE integrated into the *serW* locus is shown in Fig. S5. Notably, although the Sp15 prophage in the O157:H7 strain Sakai encodes the *stx1* gene [Hayashi T, Makino K, Ohnishi M, Kurokawa K, Ishii K *et al.* Complete genome sequence of enterohemorrhagic *Escherichia coli* O157:H7 and genomic comparison with a laboratory strain K-12. *DNA Res* 2001;8(1):11-22], "Sp15_like" prophages of clade 8 strains do not encode it. Asterisks indicate disrupted genes.

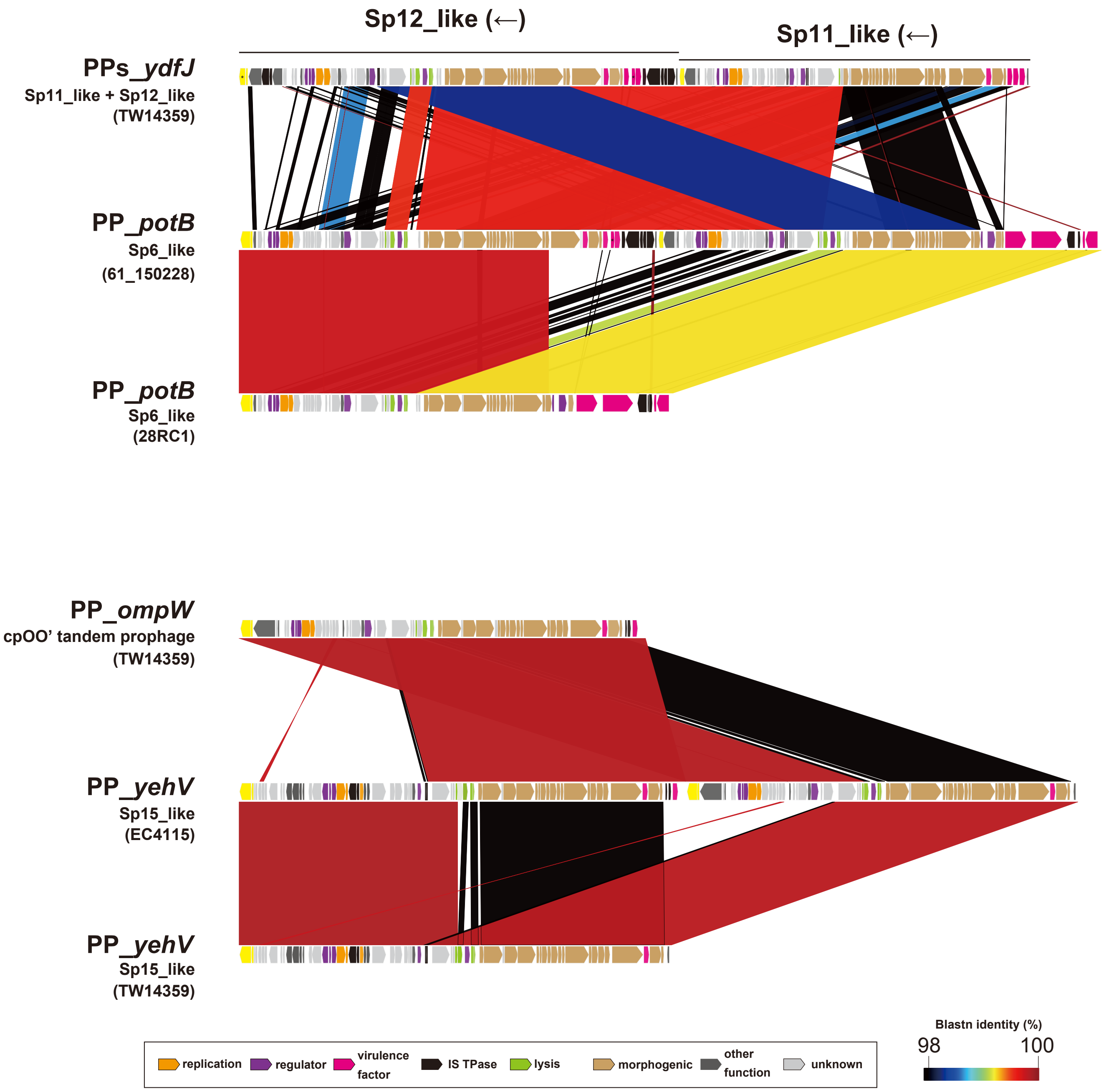

Fig. S8. An example of the PP additionally integrated in tandem (upper panel) and that of the PP inserted into another PP by homologous recombination and not by phage integrase (lower panel). Asterisks indicate disrupted genes.

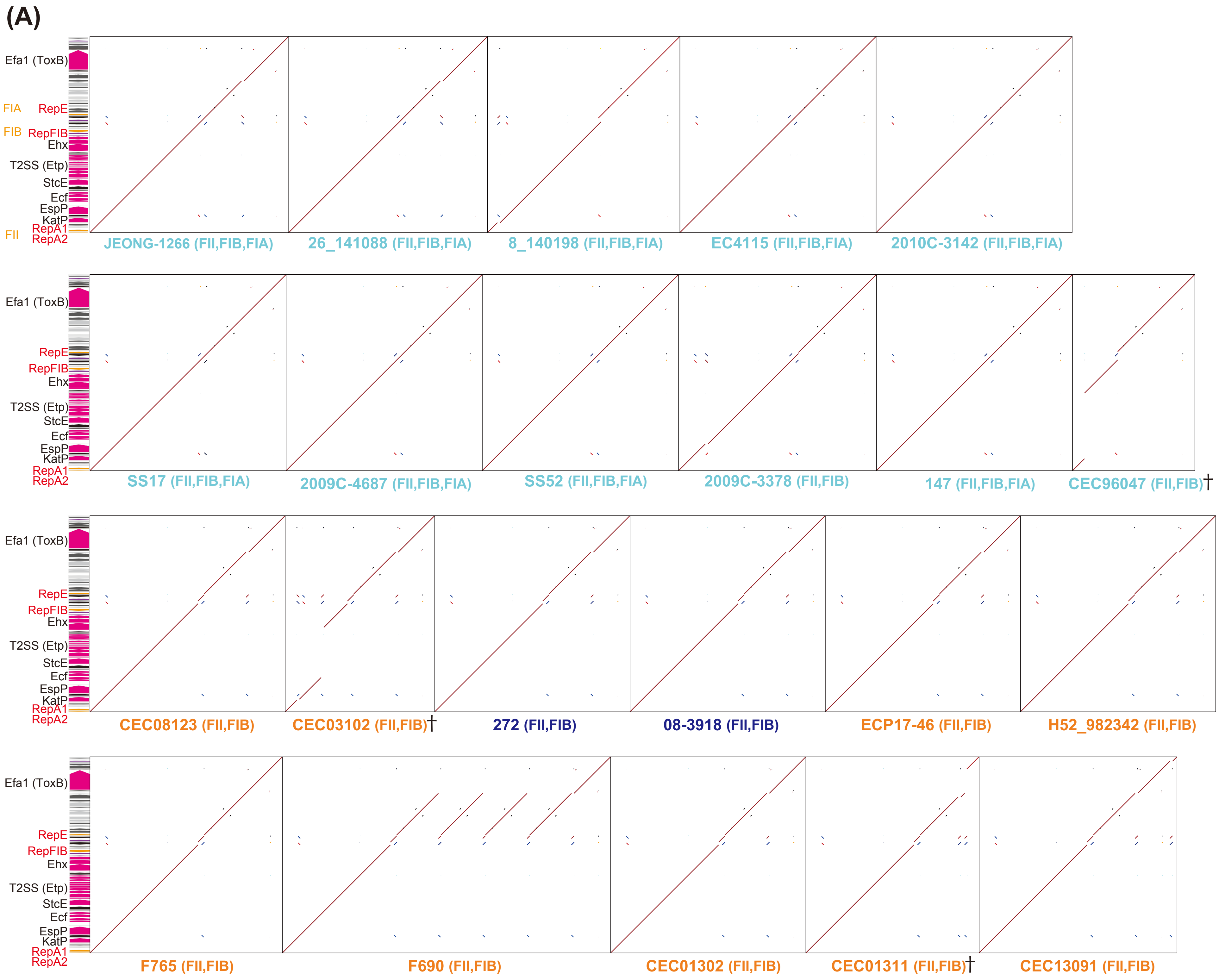
(A)
Efa1 (ToxB)
FIA
RepE
FIB
RepFIB
Ehx
T2SS (Etp)
StcE
Ecf
EspP
KatP
FII
RepA1
RepA2
JEONG-1266 (FII,FIB,FIA)
26_141088 (FII,FIB,FIA)
8_140198 (FII,FIB,FIA)
EC4115 (FII,FIB,FIA)
2010C-3142 (FII,FIB,FIA)
SS17 (FII,FIB,FIA)
2009C-4687 (FII,FIB,FIA)
SS52 (FII,FIB,FIA)
2009C-3378 (FII,FIB)
147 (FII,FIB,FIA)
CEC96047 (FII,FIB)†
CEC08123 (FII,FIB)
CEC03102 (FII,FIB)†
272 (FII,FIB)
08-3918 (FII,FIB)
ECP17-46 (FII,FIB)
H52_982342 (FII,FIB)
F765 (FII,FIB)
F690 (FII,FIB)
CEC01302 (FII,FIB)
CEC01311 (FII,FIB)†
CEC13091 (FII,FIB)

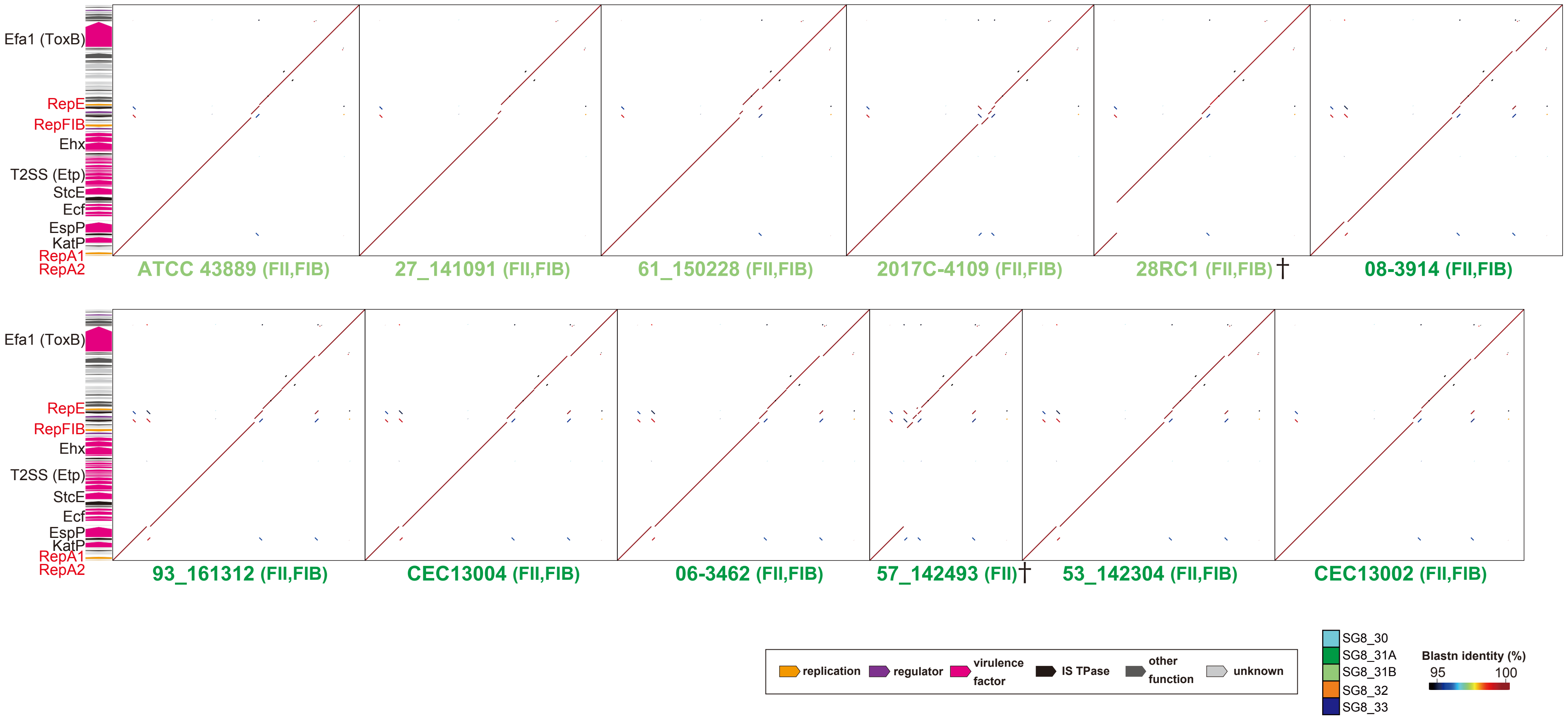

Efa1 (ToxB)
RepE
RepFIB
Ehx
T2SS (Etp)
StcE
Ecf
EspP
KatP
RepA1
RepA2
ATCC 43889 (FII,FIB)
27_141091 (FII,FIB)
61_150228 (FII,FIB)
2017C-4109 (FII,FIB)
28RC1 (FII,FIB)†
08-3914 (FII,FIB)
93_161312 (FII,FIB)
CEC13004 (FII,FIB)
06-3462 (FII,FIB)
57_142493 (FII)†
53_142304 (FII,FIB)
CEC13002 (FII,FIB)
replication
regulator
virulence factor
IS TPase
other function
unknown
SG8_30
SG8_31A
SG8_31B
SG8_32
SG8_33
Blastn identity (%)
95
100

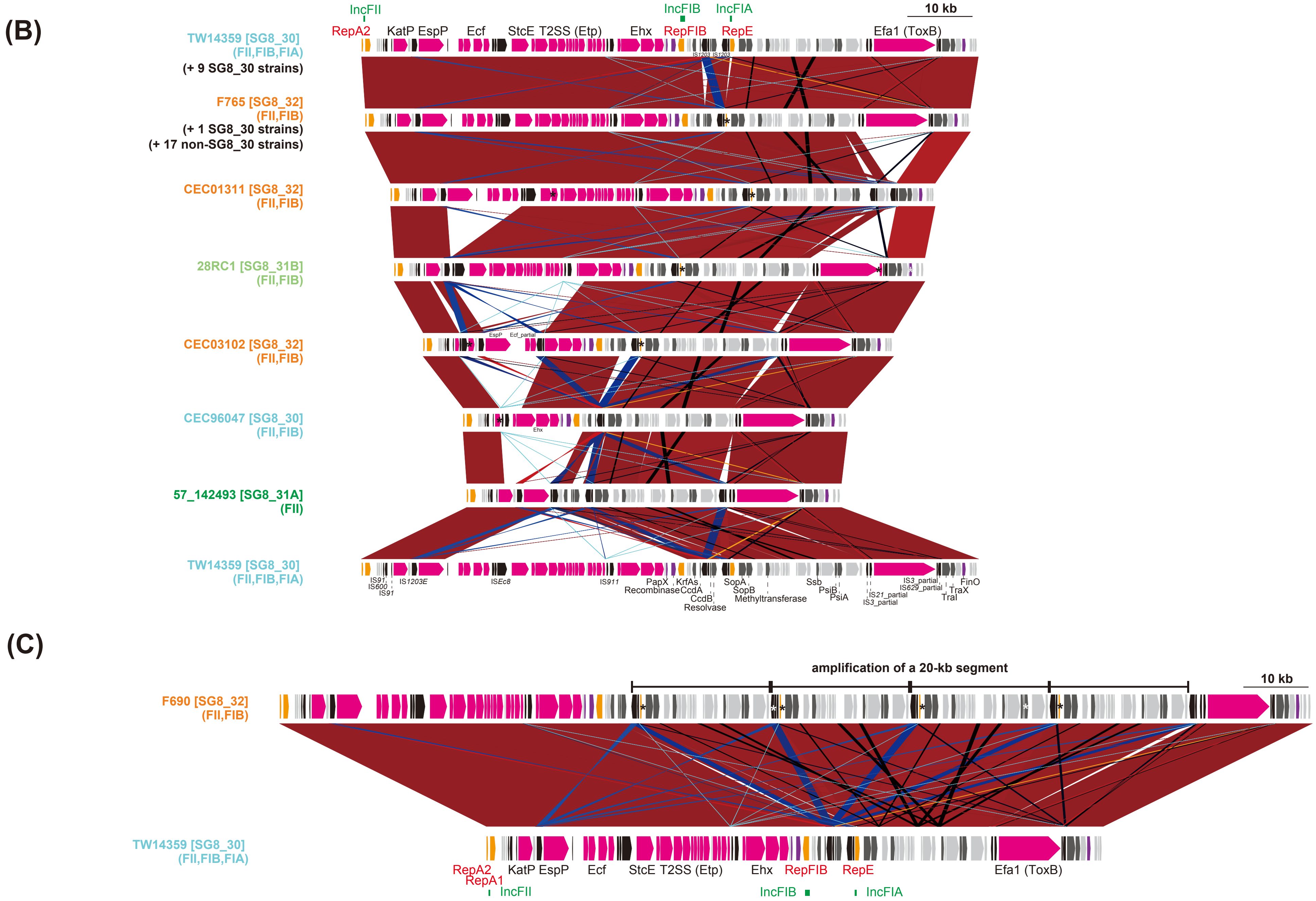

Fig. S9. Plasmids in 35 closed genomes. (A) Dot plot analyses of the pO157 virulence plasmids of each strain using the pO157 of strain TW14359 (SG8_30) as a reference (y-axis). Strain names and replicon types are colored according to the SG of each strain. (B) Structural variations of pO157. The genomic structures of representative plasmids (indicated by daggers in Fig S9A) are drawn to scale. Replication genes and replicon names are indicated in orange and green, respectively. Virulence-related genes are indicated in red along with their gene product names. Asterisks indicate disrupted genes. Note that the IncFIA replicons of the pO157 plasmids in all non-SG8_30 strains are disrupted or deleted. (C) A 150-kb-long pO157 plasmid in strain F690 (SG8_32). In this plasmid, a 20-kb segment is amplified (four times), probably via an IS*1203*-mediated mechanism.

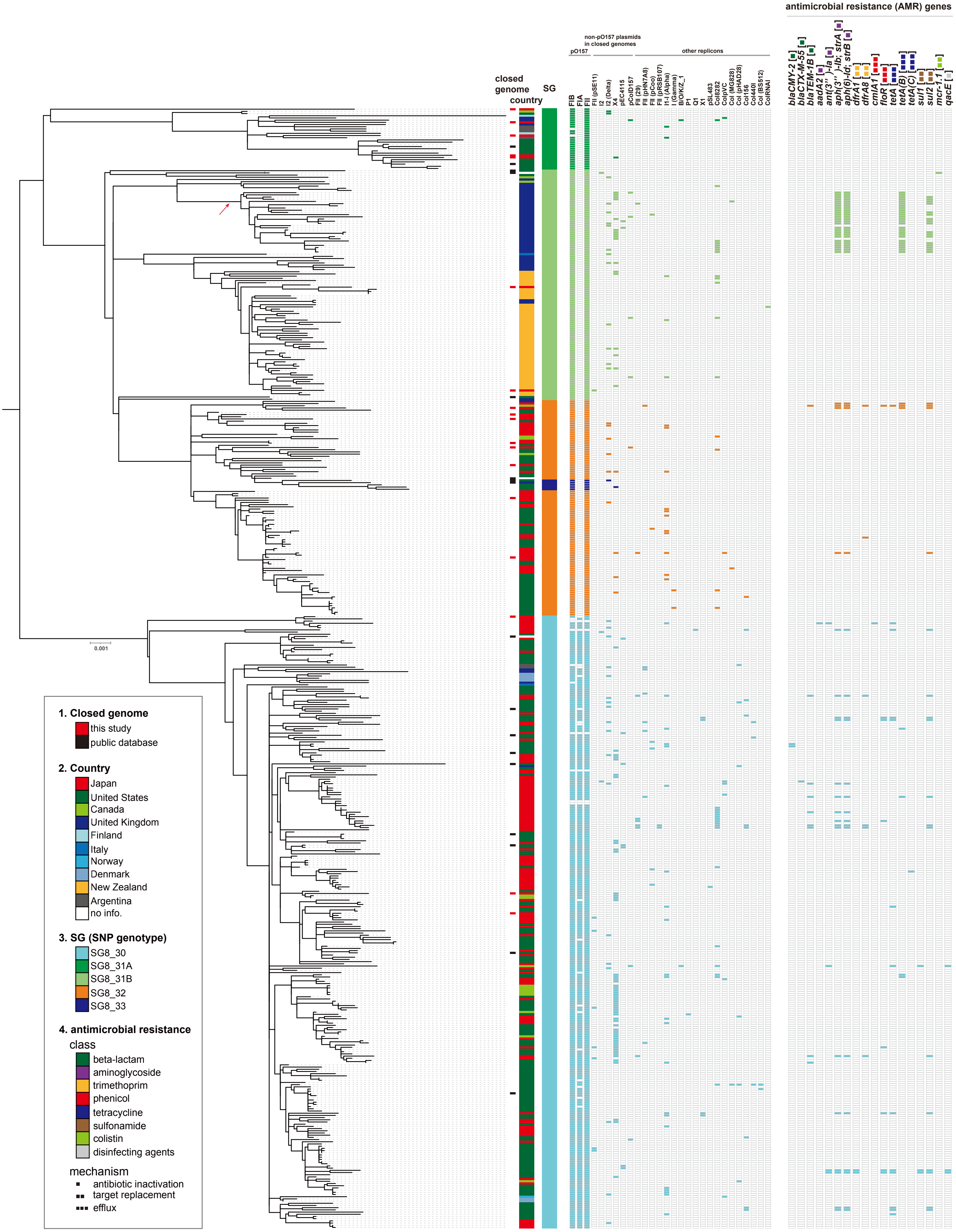

Fig. S10. Distribution of plasmid replicons and AMR genes among 510 clade 8 strains. The data are colored according to the SG of each strain.

(A)

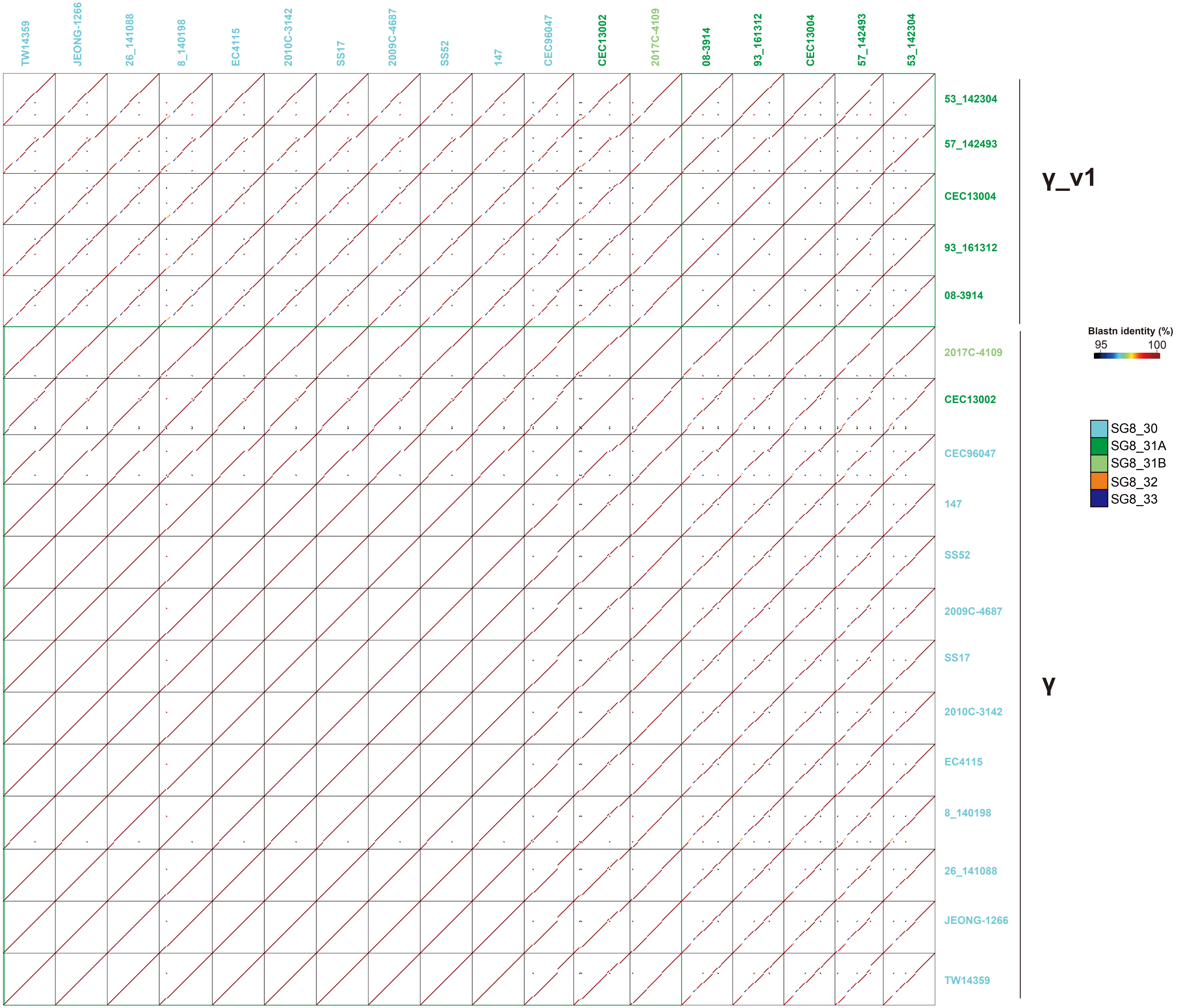
TW14359
JEONG-1266
26_141088
8_140198
EC4115
2010C-3142
SS17
2009C-4687
SS52
147
CEC96047
CEC13002
2017C-4109
08-3914
93_161312
CEC13004
57_142493
53_142304
53_142304
57_142493
CEC13004
93_161312
08-3914
2017C-4109
CEC13002
CEC96047
147
SS52
2009C-4687
SS17
2010C-3142
EC4115
8_140198
26_141088
JEONG-1266
TW14359
γ_v1
γ
Blastn identity (%)
95
100
SG8_30
SG8_31A
SG8_31B
SG8_32
SG8_33

(B)

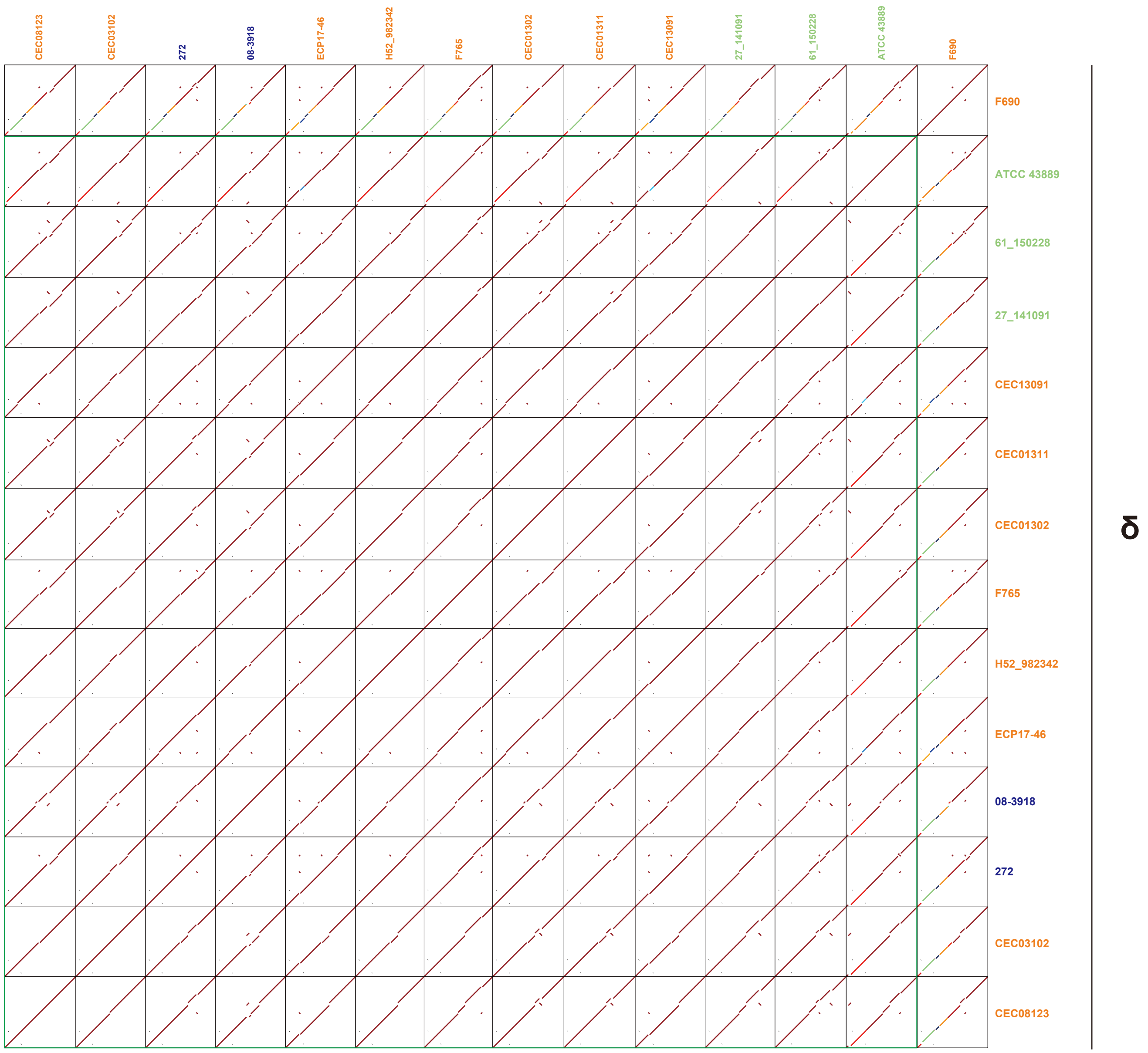

Fig. S11. All-to-all dot plot analysis of Stx2a phages of γ and γ_v1 (A) and δ (B) subtypes. Very similar Stx2a phages are framed by green squares. Strain names are colored according to their SGs.

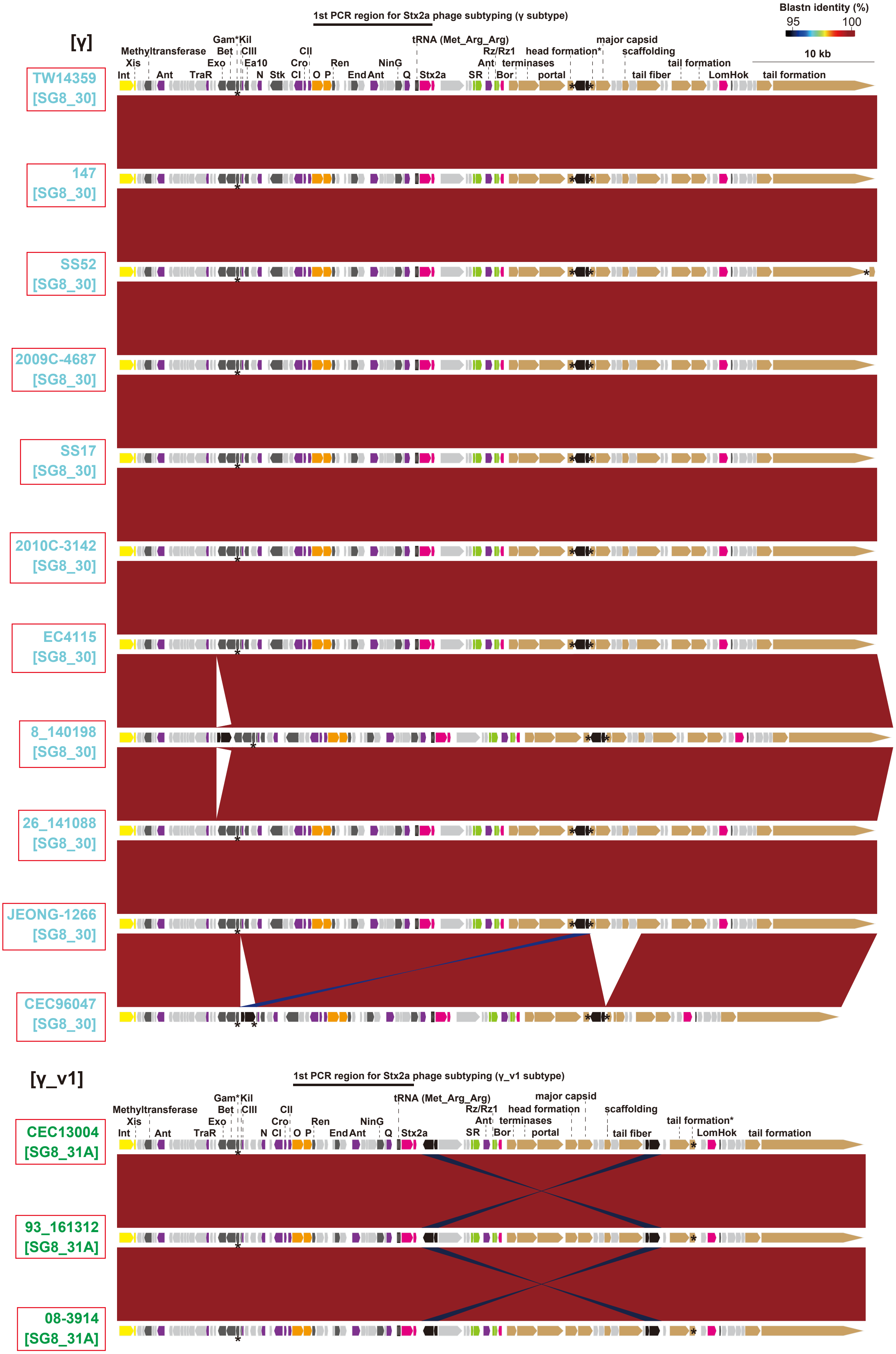
1st PCR region for Stx2a phage subtyping (γ subtype)
Blastn identity (%)
95
100
10 kb
[γ]
Int
Xis
Methyltransferase
Ant
TraR
Exo
Bet
Gam*Kil
CIII
Ea10
N
Stk
CI
Cro
CII
O
P
Ren
End
Ant
NinG
Q
tRNA (Met_Arg_Arg)
Stx2a
SR
Ant
Rz/Rz1
Bor
terminases
head formation*
portal
major capsid
scaffolding
tail fiber
tail formation
LomHok
tail formation
TW14359 [SG8_30]
147 [SG8_30]
SS52 [SG8_30]
2009C-4687 [SG8_30]
SS17 [SG8_30]
2010C-3142 [SG8_30]
EC4115 [SG8_30]
8_140198 [SG8_30]
26_141088 [SG8_30]
JEONG-1266 [SG8_30]
CEC96047 [SG8_30]
1st PCR region for Stx2a phage subtyping (γ_v1 subtype)
[γ_v1]
Int
Xis
Methyltransferase
Ant
TraR
Exo
Bet
Gam*Kil
CIII
N
CI
Cro
CII
O
P
Ren
End
Ant
NinG
Q
tRNA (Met_Arg_Arg)
Stx2a
SR
Ant
Rz/Rz1
Bor
terminases
head formation
portal
major capsid
scaffolding
tail fiber
tail formation*
LomHok
tail formation
CEC13004 [SG8_31A]
93_161312 [SG8_31A]
08-3914 [SG8_31A]

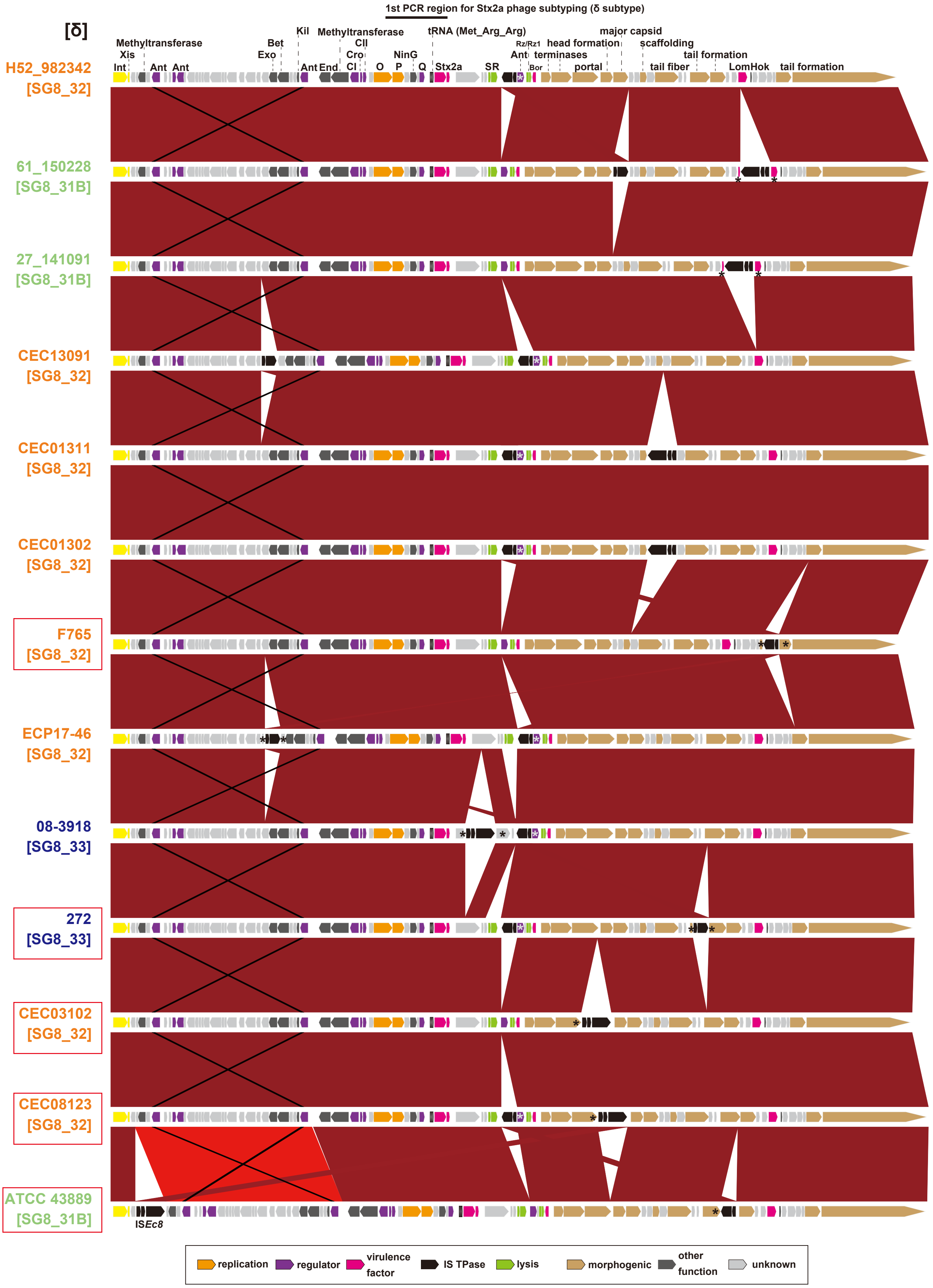

Fig. S12. Genomic structures of Stx2a phages showing high sequence similarity. See Fig. 4 in the main text for the genetic structures of two γ subtype phages in strains CEC13002 and 2017C-4109, two γ_v1 subtype phages in strains 57_142493 and 53_142304, and one δ subtype phage in strain F690. The δ subtype phage in strain ATCC 43889 (SG8_31B) contains approximately 140 SNPs compared to the other δ subtype phages, but almost all of these SNPs are located within a 2-kb region (position; 16-18 kb). Stx2a phages that contain at least one inactivated morphogenic gene are indicated by rectangles. Asterisks indicate disrupted genes.
